# HuR (Elavl1) and HuB (Elavl2) Stabilize Matrix Metalloproteinase-9 mRNA During Seizure-Induced Mmp-9 Expression in Neurons

**DOI:** 10.3389/fnins.2018.00224

**Published:** 2018-04-09

**Authors:** Katarzyna Zybura-Broda, Malgorzata Wolder-Gontarek, Magdalena Ambrozek-Latecka, Artur Choros, Agnieszka Bogusz, Joanna Wilemska-Dziaduszycka, Marcin Rylski

**Affiliations:** Department of Clinical Cytology, Centre of Postgraduate Medical Education, Warsaw, Poland

**Keywords:** HuR, HuB, hippocampus, Mmp-9, mRNA decay, mRNA stability, neuronal activation, neuronal plasticity

## Abstract

Matrix metalloproteinase-9 (Mmp-9) is involved in different general and cell-type–specific processes, both in neuronal and non-neuronal cells. Moreover, it is implicated in an induction or progression of various human disorders, including diseases of the central nervous system. Mechanisms regulating activity-driven Mmp-9 expression in neurons are still not fully understood. Here, we show that stabilization of Mmp-9 mRNA is one of the factors responsible for the neuronal activity-evoked upregulation of Mmp-9 mRNA expression in hippocampal neurons. Furthermore, we demonstrate that the molecular mechanism related to this stabilization is dependent on the neuronal seizure-triggered transiently increased binding of the mRNA stability-inducing protein, HuR, to ARE1 and ARE4 motifs of the 3′UTR for Mmp-9 mRNA as well as the stably augmented association of another mRNA-stabilizing protein, HuB, to the ARE1 element of the 3′UTR. Intriguingly, we demonstrate further that both HuR and HuB are crucial for an incidence of Mmp-9 mRNA stabilization after neuronal activation. This study identifies Mmp-9 mRNA as the first HuB target regulated by mRNA stabilization in neurons. Moreover, these results are the first to describe an existence of HuR-dependent mRNA stabilization in neurons of the brain.

## Introduction

Electrical activity is an important component of neuronal behavior. It influences multiple cellular phenomena, including gene expression, and participates in neuronal response to external stimuli. The neuronal activity-regulated protease Mmp-9 plays an important role in synaptic plasticity, learning, and memory as well as in many central nervous system (CNS) diseases, including stroke, epilepsy, schizophrenia, neurodegenerative disorders, and so on (Vafadari et al., 2016). However, the neuronal activity-dependent control of Mmp-9 expression is not well understood.

We have previously shown that the neuronal activity-dependent Mmp-9 mRNA upregulation in the hippocampal neurons is dependent on the coordinated action of YY1 and AP-1 transcription factors as well as HDAC3-related histone deacetylation (Rylski et al., 2008, 2009). Moreover, it has been suggested that miR-132 can be involved in this process (Jasinska et al., 2016). RNA-binding proteins are important regulators of neuronal gene expression as well as of CNS development and functions (Bolognani and Perrone-Bizzozero, 2008; Bryant and Yazdani, 2016). They are abundantly expressed in the brain and participate in the pathogenesis of numerous CNS diseases (Bryant and Yazdani, 2016).

We hypothesize that, in the hippocampus, mechanisms of Mmp-9 mRNA stabilization are involved in the neuronal activation-dependent upregulation of its expression and that they are driven by regulatory proteins binding to its 3′UTR region. Here, we reveal a new dimension of activity-driven regulation of Mmp-9 expression. We present for the first time that Mmp-9 mRNA expression in the brain is regulated by posttranscriptional mechanisms dependent on modulation of mRNA stability. Specifically, we show that cytoplasmic fractions of RNA-binding proteins HuR and HuB increasingly bind and stabilize hippocampal Mmp-9 mRNA in response to the pentylenetetrazole-induced neuronal activation that participates in its upregulation. Moreover, we provide evidence suggesting that both HuR and HuB actions are critically required for the occurrence of seizure-evoked Mmp-9 mRNA stabilization in the hippocampal neurons.

## Materials and methods

### Reagents

The Gibco™ Minimum Essential Medium (MEM), Gibco™ MEM Non-Essential Amino Acids, GlutaMAX, penicillin, streptomycin, Neurobasal medium, glutamine, TranscriptAid T7 High Yield transcription Kit, Pierce™ RNA 3′ End Biotynylation Kit, LightShift® Chemiluminescent RNA EMSA Kit, and HuR siRNA (Elavl1 Silencer® Select siRNA, Ambion) were purchased from Thermo Fisher Scientific, USA. Poly-d-lysine, glutamic acid, β-mercaptoethanol, cytosine-β-d-arabinofuranoside, actinomycin D, TRI Reagent, and pentylenetetrazole (PTZ) were obtained from Sigma-Aldrich, USA. Papain latex was from Worthington, USA. The electroporation Buffer R was procured from the Neon Transfection System 100 μL Kit, Invitrogen, USA.

### Animals

Adult male Wistar rats weighing approximately 200–230 g were obtained from Mossakowski Medical Research Centre Polish Academy of Sciences (Warsaw, Poland). They were individually caged in a temperature- and humidity-controlled room (23°C, ~50% humidity), on a 12/12 light/dark cycle. Food and water were available *ad libitum*. Prior to the experiments, all rats were habituated to handling for a week. The animal studies were approved by the IV Warsaw Ethical Committee on Animal Research at the Centre of Postgraduate Medical Education. All procedures on rats were conducted in accordance with the rules established by the IV Local Ethical Committee on Animal Research of the Centre of Medical Postgraduate Education on the basis of national laws. All efforts were made to minimize animal stress and sufferings.

### Pentylenetetrazole stimulation

Rats were handled and received intraperitoneal injections of saline once a day for three consecutive days before commencement of the experimental treatment. Animals were randomly divided into two groups: experimental and control. Then, rats from the experimental group received an intraperitoneal injection of PTZ (50 mg/kg of rat body weight, dissolved in physiological saline). Behavioral seizures were scored according to a modified Racine's scale (0, no behavioral changes; 1, facial movements and ear and whisker twitching; 2, myoclonic convulsions without rearing; 3, myoclonic convulsions with rearing; 4, clonic convulsion with loss of posture; and 5, generalized clonic–tonic seizures). Only animals displaying seizures and scored as 4 or 5 were used for further experiments; these animals were sacrificed 2 or 4 h after the onset of convulsions, and both hippocampi were dissected for further analyses. Animals with seizures scored as 1–3 and those which died before the appropriate time points were not included in further studies.

### Primary rat hippocampal neuronal cultures

Rat primary hippocampal neurons were obtained from neonatal (P0–P1) Wistar rats. Animals were decapitated, and both of the hippocampi were dissected out and dissociated using papain latex. Then, neuronal cells were plated at a density of 2 × 10^5^ cells/mL in Gibco™ MEM supplemented with 10% bovine calf serum, 1% Gibco™ MEM Non-Essential Amino Acids, 1% GlutaMAX, 1% sodium pyruvate, 0.3% glucose, 100 units/mL penicillin, and 0.1 mg/mL streptomycin and were maintained in a humidified incubator with 95% O_2_/5% CO_2_ at 37°C on 3.5 cm^2^/well plates that were previously coated with 50 μg poly-d-lysine/well. Then, 2–3 h after plating, MEM was replaced with Neurobasal Medium that supplemented with 2% B27 supplement, 100 units/mL penicillin, 0.1 mg/mL streptomycin, 1% glutamine, 0.5% glutamic acid, and 25 μM β-mercaptoethanol. On the third day after seeding, 5 mM cytosine-β-d-arabinofuranoside was added to the cultures to inhibit the proliferation of non-neuronal cells. To maintain the cultures, it was necessary to replace the medium containing supplements every 3–4 days.

### Transfection of the primary hippocampal neurons

DNA electroporation was done using the Neon Transfection System. Neuronal cells were isolated and dissociated (as described earlier) and washed with phosphate-buffered saline without Ca^2+^ and Mg^2+^. Then, these cells were resuspended at high cell density in Electroporation Buffer R to a final concentration of 1 × 10^7^ cells/mL. Electroporations were undertaken in a 100-μL tip containing 1 × 10^6^ cells. Optimal results of electroporation were achieved using a single 20-ms pulse at 1,400 V. Immediately after electroporation, cells were plated with high density (5 × 10^5^ cells/well) on 12-well poly-d-lysine-coated cell plates containing the Neurobasal Medium without antibiotics. After 24 h of plating, the Neurobasal Medium without antibiotics was replaced with Neurobasal Medium supplemented with 100 units/mL penicillin and 0.1 mg/mL streptomycin.

### Bicuculline treatment of the primary rat hippocampal neuronal cultures

On Day 7 of *in vitro* culture (DIV 7), neuronal cultures were treated with bicuculline (50 μM) dissolved in dimethyl sulfoxide. Bicuculline was added for 30 min and then washed away by replacing the medium three times. Cultures were placed back in the incubator for the next 3.5 h before addition of the TRI Reagent.

### Estimation of Mmp-9 MRNA half-life time (T_1/2_)

The T_1/2_ for Mmp-9 mRNA was estimated in the primary rat hippocampal neuronal cultures induced by bicuculline (50 μM) as described earlier. Four hours after bicuculline stimulation, a new medium containing actinomycin D (5 μg/mL) was added. Starting from the time of actinomycin D addition, RNA was isolated from the cultures (unstimulated as well as stimulated with bicuculline) at the following time points 0, 1, 2, 4, 6, 8, and 12 h. RNA was isolated and analyzed by reverse transcription quantitative PCR (RT-qPCR).

### RNA isolation

Total RNA was extracted from the rat hippocampi and primary rat hippocampal neuronal cultures using TRI Reagent according to the manufacturer's protocol.

### Reverse transcription quantitative PCR

RT-qPCR was carried out as described previously (Zybura-Broda et al., 2016). Primers and conditions for RT-qPCR are listed in Table 1. RT-PCR combined with subsequent agarose gel electrophoresis was undertaken as described previously (Rylski et al., 2009). The primers and conditions for RT-PCR are listed in Table 1.

**Table 1:** Primers and conditions for RT-qPCR and cloning experiments.

**Gene**	**Primers**	**Product length**	**PCR conditions**
**Reverse Transcription Quantitative PCR (RT-qPCR), Reverse Transcription PCR (RT-PCR)**			
Mmp-9	F: 5′-AAATGTGGGTGTACACAGGC-3′	309 bp	45 cycles at 95°C for 10 s, 55°C for 15 s and 72°C for 15 s
	R: 5′-TTCACCCGGTTGTGGAAACT-3′		
HuB	F: 5′- GCTCAATATGGCTTATGGAGTG-3′-3′	155 bp	45 cycles at 95°C for 10 s, 62°C for 15 s and 72°C for 15 s
	R: 5′-GCCACAGGATACTTTCGTCTGC-3′		
HuR	F: 5′-ATGAAGACCACATGGCGGAA-3′	244 bp	45 cycles at 95°C for 10 s, 60°C for 15 s and 72°C for 15 s
	R: 5′-AGCCTCAAGCCGTTCAGTGT-3′		
HuC	F: 5′-GGACATTGAATCCTGCAAG-3′	145 bp	45 cycles at 95°C for 10 s, 60°C for 15 s and 72°C for 15 s
	R: 5′-CTTGATGGTCTTGGTCTGC-3′		
18S RNA	F: 5′-CATAAACGATGCCGACTGGCG-3′	146 bp	45 cycles at 95°C for 10 s, 60°C for 15 s and 72°C for 15 s
	R: 5′-CTCCTGGTGGTGCCCTTCC-3′		
**Overexpression**			
HuB	F:5′-CATGGCGCGCCTATGACACAGGAAGAAC-3′	1,167 bp	40 cycles of 98°C for 10 s, 62°C for 15 s and 72°C for 30 s
	R:5′-CGCGAATTCTTAGGCTTTGTGCGTTTTG-3′		
HuR	F:5′-CATGGCGCGCCTATGTCTAATGGTTATG-3′	1,013 bp	40 cycles of 98°C for 10 s, 58°C for 15 s and 72°C for 30 s
	R:5′-CGCGGTACCGCGTGAGCGAGTTATTTGTG-3′		
HuC	F:5′-CATGGCGCGCCTATGGTCACTCAGATAC-3′	1,125 bp	40 cycles of 98°C for 10 s, 60°C for 15 s and 72°C for 30 s
	R:5′-CGCGAATTCTCAGGCCTTGTGCTGCTTG-3′		

*F, forward; R, reverse*.

### Protein lysates

Rat hippocampal cytoplasmic lysates were prepared as described previously (Rylski et al., 2008).

### *In vitro* RNA degradation assay

RDA protocol was adapted from Akool and colleagues (Akool et al., 2003). RNA used for RDA was extracted from the hippocampi of rats at 4 h after the PTZ-induced seizures because, at this time point, there would be an abundance of Mmp-9 mRNA. Thereafter, 20 μg RNA (obtained each time from the same sample) was added to 130 μg of cytoplasmic protein extracts derived from rat hippocampi isolated from unstimulated rats (control) and treated animals at 2, 4, and 6 h after the PTZ treatment. Mixtures of RNA and protein extracts were incubated for different durations (0, 30, 60, or 120 min). After incubation, RNA was extracted using TRI Reagent according to the manufacturer's protocol, and analyzed quantitatively by RT-qPCR. Primers and conditions used for RT-qPCR are listed in Table 1.

### Western blot

Western blotting was conducted as described previously (Rylski et al., 2008). Detailed information about antibodies used therein is presented in Table 2.

**Table 2:** Antibodies for WB, neutralization, and REMSA Supershift.

**Antibody**	**Supplier, catalog number**	**Method^a^**
HuB (N-15)	Santa Cruz Biotechnology, sc-5982	WB (1:300), REMSA Supershift, neutralization
HuC (G-15)	Santa Cruz Biotechnology, sc-5981	REMSA Supershift, neutralization
HuD (L-20)	Santa Cruz Biotechnology, sc-5979	REMSA Supershift
HuR (3A2)	Santa Cruz Biotechnology, sc-5261	WB (1:1,000), REMSA Supershift, neutralization
GAPDH	Millipore, MAB374	WB (1:5,000)
TBP	Pierce (Thermo Scientific), MA5-14739	WB (1:1,000)
normal mouse IgG	Santa Cruz Biotechnology, sc-2025	REMSA Supershift, neutralization
normal goat IgG	Santa Cruz Biotechnology, sc-2028	REMSA Supershift, neutralization

a *In parentheses, dilutions used for WB are shown*.

*WB, Western Blot; REMSA, RNA electrophoretic mobility shift assay*.

### RNA electrophoretic mobility shift assay and REMSA supershift analysis

Single-stranded RNA oligonucleotides were prepared using TranscriptAid T7 High Yield transcription Kit (Table 3). T4 RNA ligase was used to conjugate a single nucleotide analog (biotinylated cytidine (bis)phosphate) to the 3′-terminus of the RNA strand (Pierce™ RNA 3′ End Biotynylation Kit). Biotinylated RNA probes were incubated with 10 μg of hippocampal cytoplasmic extracts at room temperature for 30 min in a buffer containing 10 mM HEPES (7.3), 20 mM KCl, 1 mM MgCl_2_, 1 mM DTT, and 5% glycerol. To reduce nonspecific bindings, 100 ng/mL tRNA was added. Each reaction was carried out at a final volume of 20 μl. Supershift analyses were conducted by the addition of 2 μg of an appropriate antibody to the reaction mixture (Table 2), followed by overnight incubation at 4°C. Next, biotinylated RNA probes were added, and further incubated for 30 min at room temperature. RNA–protein and RNA-protein–antibody complexes were separated by electrophoresis in 8% non-denaturing polyacrylamide gels in Tris–borate–EDTA buffer. The RNA–protein and RNA–protein–antibody complexes were visualized by LightShift® Chemiluminescent RNA EMSA Kit. The gene-specific oligonucleotides used in these experiments are summarized in Table 3.

**Table 3:** Oligonucleotides used in REMSA and REMSA Supershift experiments.

**Oligonucleotide**	**Sequence**
UTR-ARE-1	5′-CCCUUUU**AUUUA**UUAUGUAUG-3′
UTR-ARE-2	5′-ACAUGU**AUUUA**ACCUAUAGAA-3′
UTR-ARE-3	5′-CAUCAAAC**AUUUA**UUGUGAGC-3′
UTR-ARE-4	5′-CAGAGGA**AUUUA**UUGGAUGUU-3′

*The oligonucleotides are derived from the region encompassing the AUUUA-rich motifs localized in the rat Mmp-9 mRNA 3′UTR. The consensus sequences are indicated in boldface*.

### HuR, HuB, or HuC overexpression or silencing

Rat HuR, HuC, or HuB full-length coding sequences were amplified from the rat hippocampal cDNA library and cloned into the AscI/KpnI site of the GW1 vector (the vector was a gift from Prof. J. Jaworski, International Institute of Molecular and Cell Biology, Warsaw, Poland). HuR siRNA was purchased from Ambion (Elavl1 Silencer® Select siRNA). Constructs expressing siRNA for HuB were obtained by inserting the following oligonucleotides specific to the 340–358 bp region of the rat HuB at the BglII/HindIII site of pSUPER: 5′-GATCCCCGTGAACTACATTGATCCCATTCAAGAGATGGGATCAATGTAGTTCACTTTTTGGAAA-3′ 5′-AGCTTTTCCAAAAAGTGAACTACATTGATCCCATCTCTTGAATGGGATCAATGTAGTTCACGGG-3′.

Constructs expressing siRNA for HuC were obtained by inserting the following oligonucleotides specific to the 116–134 bp region of the rat HuC at BglII/HindIII site of pSUPER:

5′-GATCCCCCCAACCTCATCGTCAACTATTCAAGAGATAGTTGACGATGAGGTTGGTTTTTGGAAA-3′.

5′-AGCTTTTCCAAAAACCAACCTCATCGTCAACTATCTCTTGAATAGTTGACGATGAGGTTGGGGG-3′.

### HuR, HuB, or HuC neutralization

The impact of HuR, HuB, or HuC on the degradation of Mmp-9 mRNA was tested by the addition of neutralizing anti-HuR, anti-HuB and anti-HuC antibodies to samples comprising cytoplasmic extracts obtained from the unstimulated (control) and PTZ-treated (2, 4, and 6 h after the onset of seizures) rat hippocampi. We added 0.4 μg antibody to 130 μg cytoplasmic extracts and the mixture was pre-incubated at room temperature for 1 h before the addition of the rat hippocampal RNA (20 μg). RNA used for neutralization experiments was extracted from the hippocampi of rats sampled 4 h after the PTZ-induced seizures. To exclude any nonspecific inhibitory effects induced by the serum, normal isotype sera were tested in parallel. Mixtures of antibody, RNA, and the protein extracts were incubated for 2 h. Then, RNA was extracted using TRI Reagent and analyzed quantitatively by RT-qPCR. Primers and conditions used for RT-qPCR are listed in Table 1. Detailed information about the antibodies used is presented in Table 2.

### Statistical analyses

The Kolmogorov–Smirnov and Shapiro–Wilk tests were used to validate the assumption of normality. Statistical significance was determined using a nonparametric, Mann–Whitney *U*-test (to compare two groups), or the Kruskal–Wallis test with Dunn's *post hoc* test (for three or more groups) for data with non-normal distribution. Parametric Student's *t*-test (to compare two groups) or one-way ANOVA with Tukey's *post hoc* pairwise test (for three or more groups) was used for data with normal distribution. Data from RNA degradation assay and Mmp-9 mRNA half-life time were tested using two-way ANOVA with Tukey or Bonferroni post hoc tests. Results are shown as mean ± SEM. Differences were considered significant at *p* < 0.05. Descriptive and analytical statistical analysis with parametric and non-parametric tests was conducted using GraphPad Prism 7 (GraphPad Software, Inc., La Jolla, CA).

## Results

### Mmp-9 MRNA is stabilized in response to neuronal activation in the hippocampus

**Figure 1:**
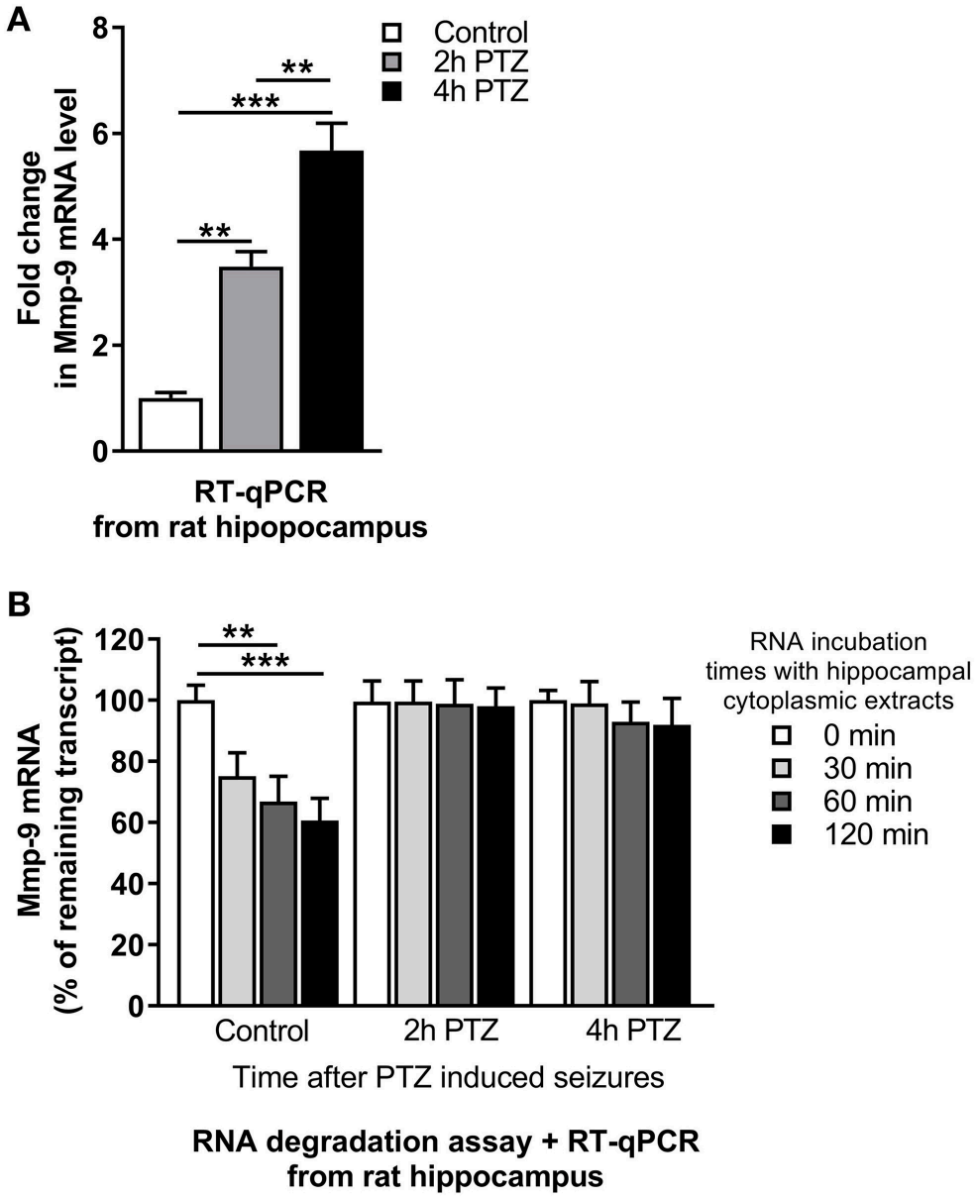
mRNA stabilization is involved in Mmp-9 mRNA upregulation after seizure-induced neuronal activation. **(A)** Mmp-9 mRNA accumulates after PTZ-induced seizures in the rat hippocampus. Mmp-9 mRNA increased progressively after PTZ-evoked neuronal activation in the rat hippocampus *in vivo*. For each analysis, equal amounts of RNA samples isolated from unstimulated (control) and PTZ-treated (2 and 4 h after the onset of seizures) rat hippocampi were used. Mmp-9 expression was normalized against 18S rRNA expression. Data are presented as fold change in mRNA expression. One-way ANOVA was applied followed by Tukey's multiple comparisons test. Values are mean ± SEM (**p < 0.01; ***p < 0.001; *n* = 4). **(B)** Mmp-9 mRNA is significantly stabilized in response to PTZ-induced seizures in the rat hippocampus. A degradation assay of Mmp-9 mRNA was conducted using equal amounts of RNA isolated from the rat hippocampus and incubated with cytoplasmic protein lysates obtained from unstimulated (control) and PTZ-treated (2 and 4 h after the onset of seizures) rat hippocampi. The results are presented as a percentage of time zero. Mmp-9 expression was normalized against 18S rRNA expression. Two-way ANOVA followed by Tukey's multiple comparisons test. Values are mean ± SEM (**p < 0.01; ***p < 0.001; *n* = 4).

To evaluate changes of Mmp-9 mRNA after neuronal activation, we isolated RNA from the unstimulated as well as PTZ-stimulated (2 and 4 h after PTZ-induced seizures) rat hippocampi and analyzed Mmp-9 mRNA content by RT-qPCR. Our results demonstrated the occurrence of substantial neuronal seizure-dependent upregulation of Mmp-9 mRNA expression (Figure 1A).

To check whether the Mmp-9 mRNA stabilization can be induced by neuronal activation, we measured the rate of Mmp-9 mRNA degradation in the hippocampus by an RNA degradation assay (RDA). We mixed cytoplasmic protein lysates isolated from the unstimulated as well as PTZ-stimulated (2 and 4 h after PTZ-induced seizures) rat hippocampi with equal amounts of rat hippocampal RNA and incubated the samples for 0, 30, 60, and 120 min. We found that Mmp-9 mRNA underwent gradual degradation in the unstimulated hippocampus (Figure 1B). In contrast, in the activated hippocampi, we observed considerably enhanced Mmp-9 mRNA stabilization (Figure 1B). This indicates that Mmp-9 mRNA is significantly stabilized in response to PTZ-evoked neuronal activation in the rat hippocampus. Altogether, the results suggest that mRNA stabilization mechanisms are involved in Mmp-9 mRNA upregulation after neuronal activation.

### Hippocampal cytoplasmic protein complexes bind *in vitro* to ARE1 and ARE4 motifs of Mmp-9 mRNA 3′UTR, and these interactions are upregulated in response to PTZ-evoked neuronal activation

Adenylate-uridylate-rich elements (AREs) of mRNA 3′-UTRs are one of the main mRNA-stabilizing motifs found in mammalian mRNAs (Chen and Shyu, 1995). It has been shown previously that there are four ARE sites in the rat Mmp-9 mRNA 3′UTR, localized at 380–384 bp (ARE1), 415–419 bp (ARE2), 564–568 bp (ARE3), and 622–626 bp (ARE4) (Akool et al., 2003). To investigate the possibility and dynamics of hippocampal cytoplasmic protein binding to the ARE1–4 sequences in response to PTZ-induced seizures, we conducted the RNA Electrophoretic Mobility Shift Assay (REMSA). We incubated biotinylated RNA probes representing four different ARE regions (ARE1, ARE2, ARE3, or ARE4) of the rat Mmp-9 mRNA 3′UTR with cytoplasmic protein lysates isolated from the unstimulated as well as PTZ-stimulated (2 and 4 h after PTZ-induced seizures) rat hippocampi. Additionally, as a control of binding specificity, we included samples obtained after incubation of cytoplasmic extracts collected from PTZ-treated (4 h after PTZ-induced seizures) rat hippocampi with biotinylated RNA probes derived from ARE1, ARE2, ARE3, or ARE4 sites of the rat Mmp-9 mRNA 3′UTR and mixed with 250× or 500× excess volumes of competitors (the corresponding unlabeled ARE probes).

**Figure 2:**
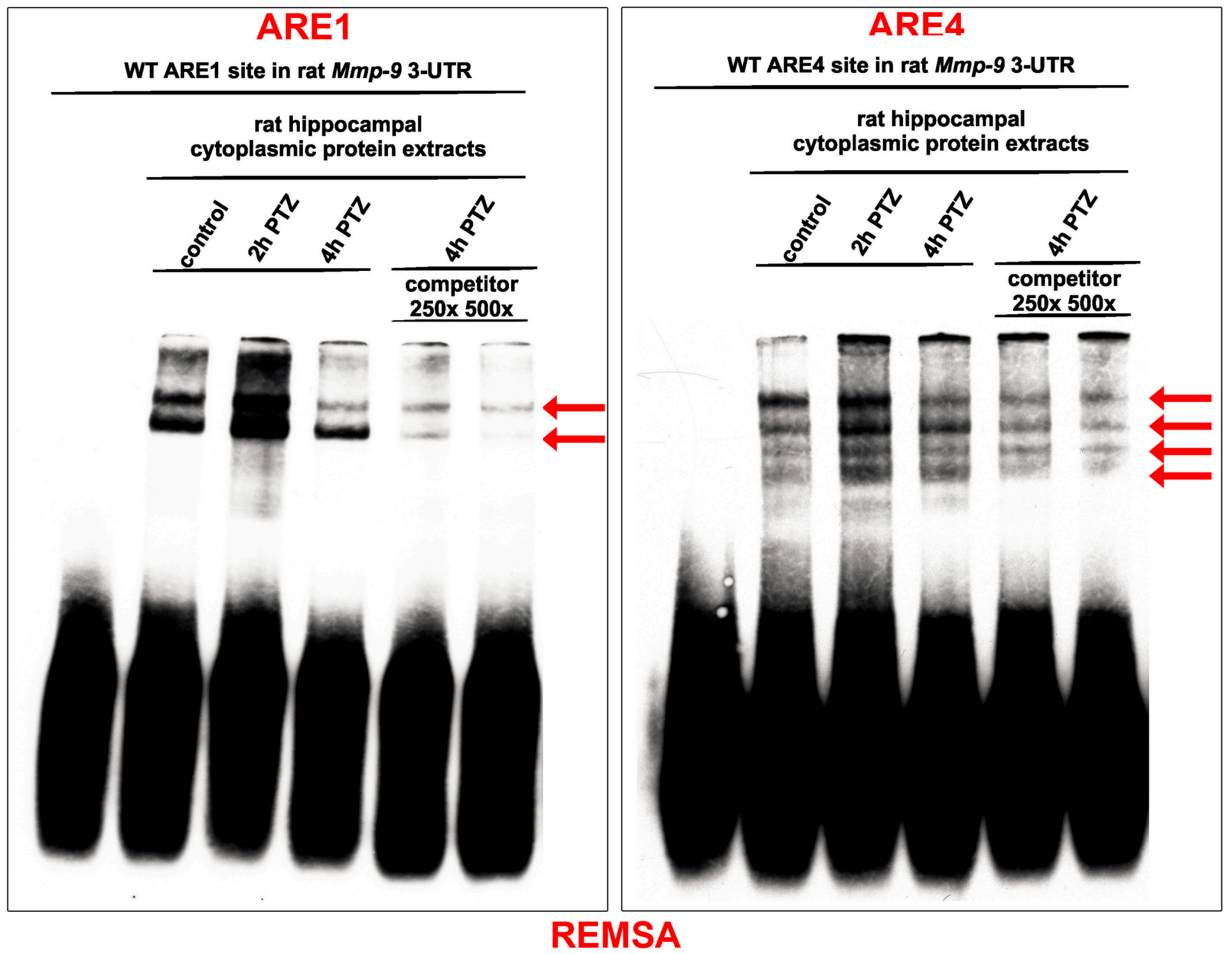
Hippocampal cytoplasmic protein complexes bind *in vitro* to ARE1 and ARE4 motifs of the Mmp-9 mRNA 3′UTR, and these interactions are increased in response to PTZ-evoked neuronal activation. The protein–RNA interactions were analyzed by REMSA. Biotynylated RNA probes derived from ARE1 or ARE4 sites of the rat Mmp-9 mRNA 3′UTR were incubated with equal amounts of cytoplasmic protein lysates isolated from the unstimulated as well as PTZ-stimulated (2 and 4 h after PTZ-induced seizures) rat hippocampi. Additionally, as a control of binding specificity, we included samples obtained by the incubation of cytoplasmic extracts collected from the PTZ-treated (4 h after PTZ) rat hippocampi with biotynylated RNA probes derived from ARE1 or ARE4 sites of the rat Mmp-9 mRNA 3′UTR and mixed with 250× or 500× excess of competitors (the corresponding unlabeled ARE probes). The specifically shifted bands are marked by arrows. Representative images are shown.

Consequently, we observed cytoplasmic protein interactions with ARE1 and ARE4, but not with ARE2 and ARE3, motifs of the rat Mmp-9 mRNA 3'UTR (Figure 2 and Figure S1). In the ARE1 and ARE4 samples, we detected the formation of two and four specific bands, respectively, and their credibility was confirmed by the gradual disappearance of band signal in competition experiments (Figure 2 and Figure S1). Additionally, we found that these specific interactions were increased in samples collected 2 h after PTZ-induced seizures (Figure 2).

### PTZ-induced neuronal activation increases binding of HuR and HuB to rat Mmp-9 MRNA 3′UTR in the rat hippocampus

**Figure 3:**
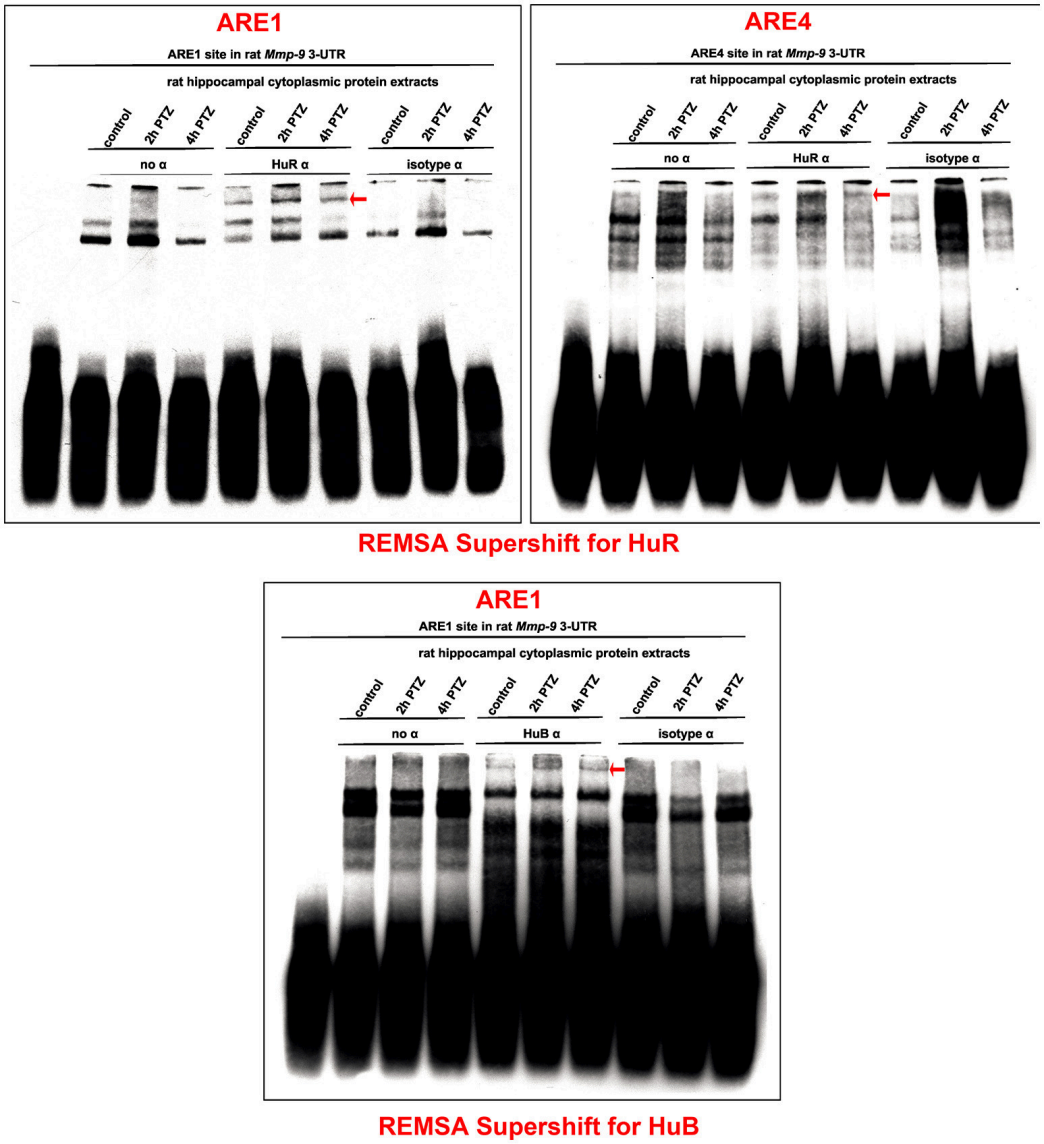
PTZ-induced neuronal activation transiently increases interactions of HuR with ARE1 and ARE4 motifs and stably stimulates HuB binding to the ARE1 site of the rat Mmp-9 mRNA 3′UTR in the rat hippocampus. The interactions of HuR and HuB with Mmp-9 mRNA were studied by REMSA Supershift. Biotinylated RNA probes representing ARE1 or ARE4 of the rat Mmp-9 mRNA 3′UTR were incubated with anti-HuR or anti-HuB antibody and with cytoplasmic protein lysates isolated from the unstimulated as well as PTZ-stimulated (2 and 4 h after PTZ-induced seizures) rat hippocampi. In control reactions, no antibody or normal isotype antibody was added to the incubates, consisting of the ARE motif and cytoplasmic protein lysate. Supershifted bands are indicated by red arrows. Representative images are shown.

Hu proteins (HuB, HuC, HuD, and HuR) are the main mRNA stabilizers binding to AREs (Peng et al., 1998; Brennan and Steitz, 2001). To investigate Hu–Mmp-9 mRNA interactions during the PTZ-evoked neuronal activation, we conducted REMSA Supershift experiments with a focus on HuB, HuC, HuD, or HuR. We incubated specific antibodies directed against HuB, HuC, HuD, or HuR with biotinylated RNA probes representing four different ARE regions (ARE1, ARE2, ARE3, or ARE4) of the rat Mmp-9 mRNA 3′UTR and with cytoplasmic protein lysates isolated from the unstimulated as well as PTZ-stimulated (2 and 4 h after PTZ-induced seizures) rat hippocampi (Figure 3 and Figures S2–S6). In control samples, no antibody or a normal isotype antibody was added to incubates composed of ARE probes and cytoplasmic protein lysates. We revealed the occurrence of supershifts in samples containing the ARE1 or ARE4 probe incubated with anti-HuR (Figure 3) as well as the ARE1 probe incubated with anti-HuB (Figure 3). Interactions of HuR with ARE1 and HuR with ARE4 were transiently increased at 2 h after the PTZ-induced seizures, and then reduced at 4 h after the seizures (Figure 3). The interaction of HuB with ARE1 was stably induced by PTZ stimulation (Figure 3). No supershift was observed in samples incubated with anti-HuC (Figure S5) and anti-HuD (Figure S6). Together, these data suggest that the binding of mRNA-stabilizing proteins to the rat Mmp-9 mRNA 3′UTR increases (transiently for HuR and stably for HuB) after PTZ-induced seizures in the rat hippocampus. Moreover, the results of REMSA Supershift confirm our REMSA data by showing that neuronal activation evokes elevated binding of the hippocampal Hu proteins to ARE1 and ARE4, but not to ARE2 and ARE3, motifs of the rat Mmp-9 mRNA 3′UTR.

### HuR and HuB are expressed in the cytoplasm of the unstimulated and PTZ-activated rat hippocampal cells

**Figure 4:**
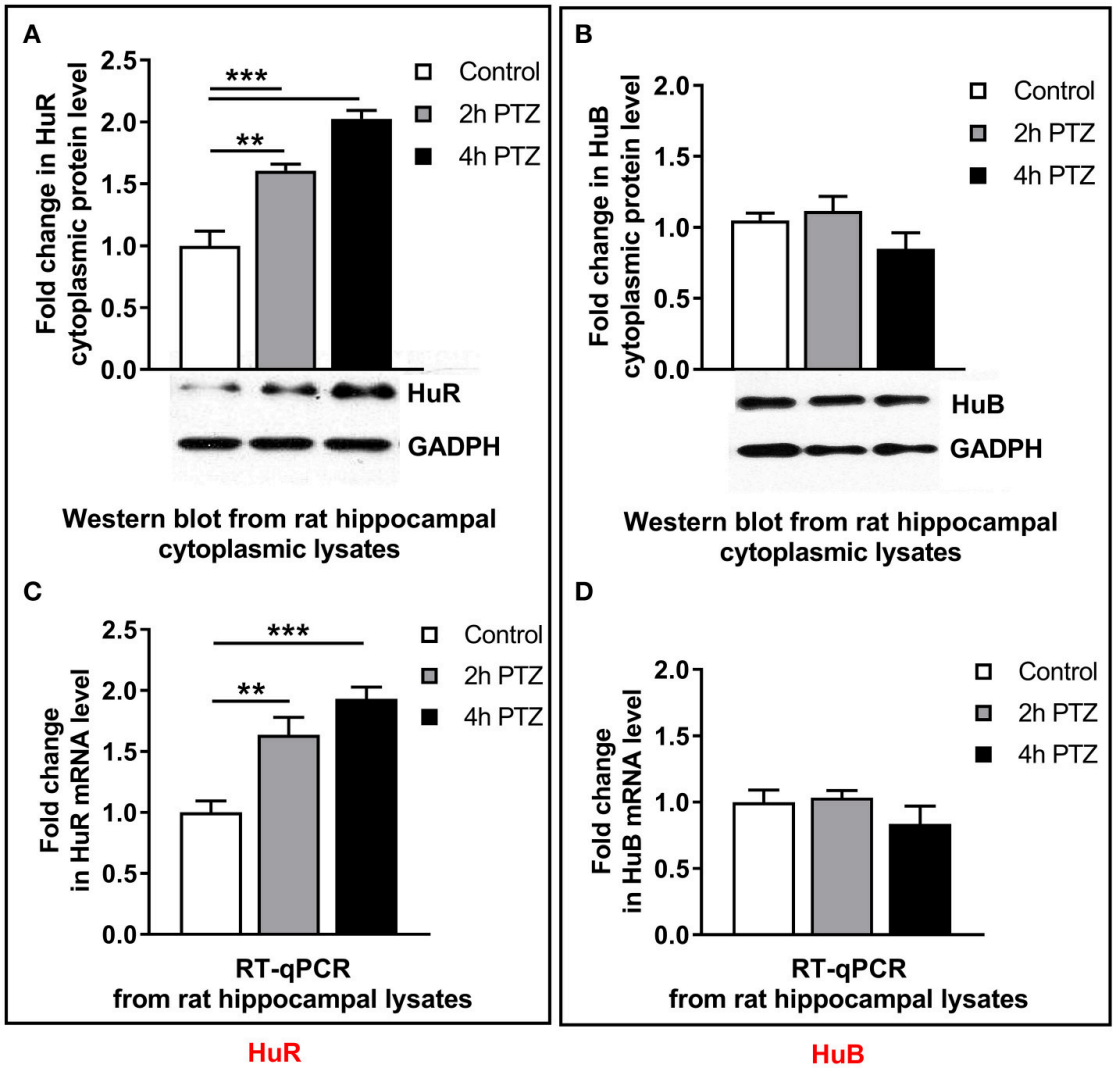
After neuronal activation in the rat hippocampus, the cytoplasmic HuR and HuR mRNA are progressively upregulated, whereas the cytoplasmic HuB and HuB mRNA are stably expressed. We administered 50 mg/kg of PTZ intraperitioneally. Rats were sacrificed at 2 and 4 h after the onset of the seizures. For each analysis, equal amounts of the cytoplasmic protein extracts or RNA isolated from the unstimulated (control) and PTZ-treated (2 and 4 h after the onset of seizures) rat hippocampi were used and analyzed by Western blotting or qRT-PCR, respectively. **(A)** HuR accumulates progressively in the hippocampal cell cytoplasm after PTZ-evoked neuronal activation. GAPDH was used as a loading control. Representative cropped Western blots are shown. Graph data is presented as fold change in the protein expression. One-way ANOVA was followed by Tukey's multiple comparisons test. Values are mean ± SEM (**p < 0.01; ***p < 0.001; *n* = 5). **(B)** HuB is stably expressed in the hippocampal cell cytoplasm after PTZ-evoked neuronal activation. GAPDH was used as a loading control. Representative cropped Western blots are shown. Graph data is presented as fold change in the protein expression. Kruskal–Wallis test was followed by Dunn's multiple comparisons test. Values are mean ± SEM (*n* = 5). **(C)** HuR mRNA accumulates progressively after PTZ-evoked neuronal activation in the rat hippocampus. HuR mRNA expression was normalized against 18S rRNA expression. Data are presented as fold change in mRNA expression. One-way ANOVA was followed by Tukey's multiple comparisons test. Values are mean ± SEM (*p < 0.05; **p < 0.01; ***p < 0.001; *n* = 5). **(D)** HuB mRNA is stably expressed after PTZ-evoked neuronal activation in the rat hippocampus. HuB mRNA expression was normalized against 18S rRNA expression. Data are presented as fold change in mRNA expression. Kruskal–Wallis test was followed by the Dunn's multiple comparisons test. Values are mean ± SEM (*n* = 5).

To confirm the possibility of HuR–Mmp-9 mRNA and HuB–Mmp-9 mRNA interactions, we used Western blotting to check whether HuR and HuB are expressed in cytoplasmic lysates of the unstimulated as well as PTZ-activated rat hippocampi. We found that HuR and HuB were detectably expressed in cytoplasmic extracts of the unstimulated rat hippocampus (Figures 4A,B). Moreover, HuR cytoplasmic expression was gradually augmented after PTZ-induced seizures, whereas the cytoplasmic HuB was stably expressed after neuronal activation in the rat hippocampus (Figure 4A,B). Similarly, RT-qPCR results showed that HuR and HuB mRNAs were expressed in the unstimulated rat hippocampus, whereas, after the PTZ-induced seizures, HuR mRNA was upregulated and HuB mRNA remained stably expressed (Figure 4C,D).

### Mmp-9 mRNA is upregulated and robustly stabilized after bicuculline-evoked activation in the hippocampal neurons

**Figure 5:**
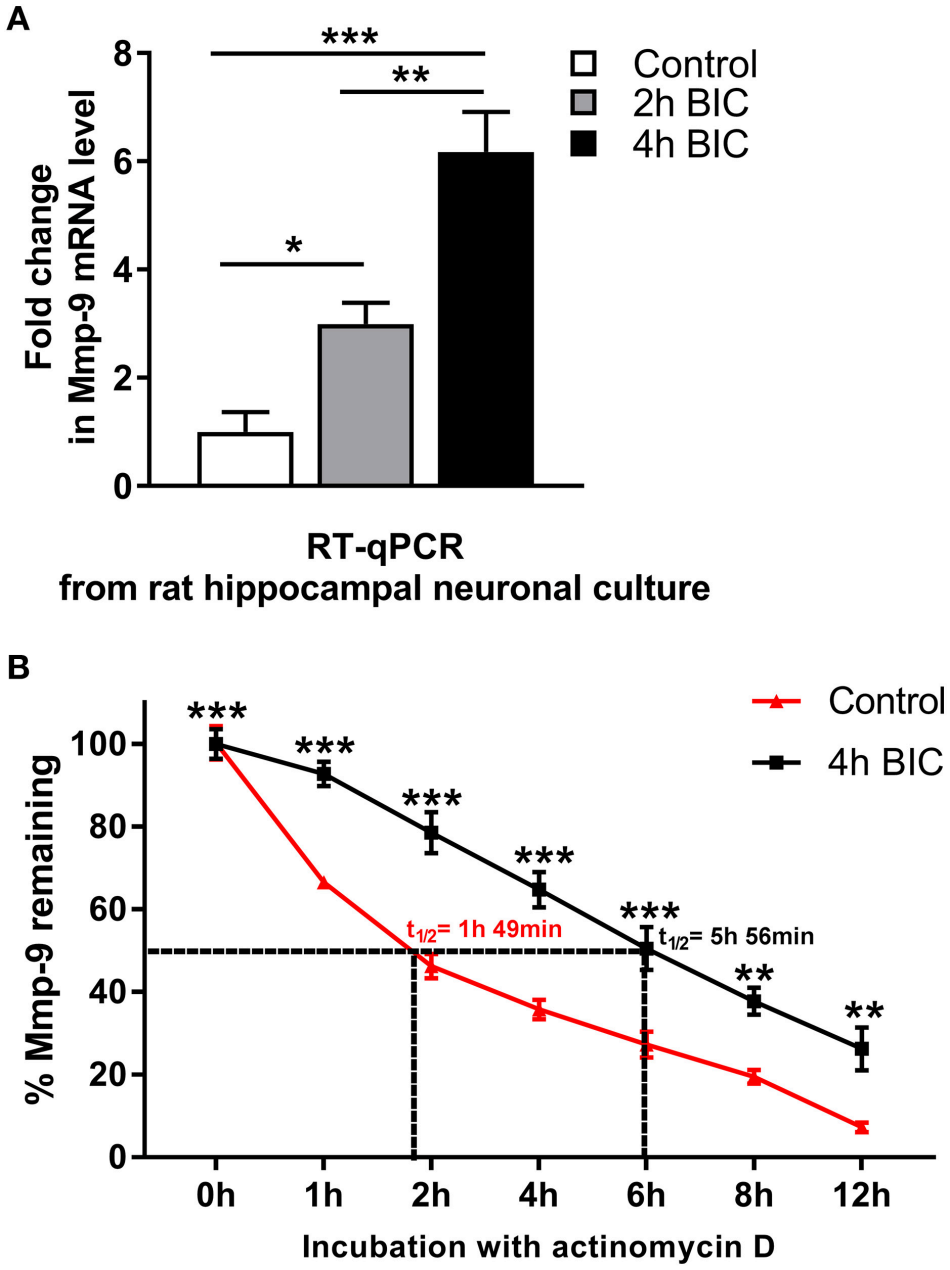
Bicuculline-evoked neuronal activation triggers upregulation and stabilization of Mmp-9 mRNA in hippocampal neurons. **(A)** Mmp-9 mRNA accumulates in primary rat hippocampal neuronal cultures after neuronal activation. On Day 7 *in vitro*, neuronal cultures were treated with bicuculline (50 μM). For each analysis, equal amounts of RNA samples isolated from the unstimulated (control) and activated (2 and 4 h after bicuculline) cultures were used. Mmp-9 expression was normalized against 18S rRNA expression. One-way ANOVA was followed by Tukey's multiple comparisons test. Values are mean ± SEM (*p < 0.05; **p < 0.01; ***p < 0.001; *n* = 6). **(B)** Bicuculline treatment strongly extends the Mmp-9 mRNA half-life in primary hippocampal neurons. On Day 7 *in vitro*, actinomycin D (5 μg/mL) was added to the medium of the unstimulated (control) and activated (4 h after bicuculline) hippocampal neuronal cultures for 30 min. Then, RNA was isolated from the cultures at 0, 1, 2, 4, 6, 8, and 12 h after administration of actinomycin D, and Mmp-9 mRNA expression was evaluated by qRT-PCR. Mmp-9 mRNA expression was normalized against 18S rRNA expression. Results are presented as a percentage of the remaining Mmp-9 mRNA transcripts (the amount of Mmp-9 mRNA at Time 0 is assumed to be 100%). Two-way ANOVA followed by Bonferroni's multiple comparisons test. Values are mean ± SEM (**p < 0.01; ***p < 0.001; *n* = 4).

Next, using the primary hippocampal neuronal cultures, we evaluated whether Mmp-9 mRNA is stabilized and upregulated in hippocampal neuronal cells in response to bicuculline-evoked activation. Bicuculline is a GABA A receptor antagonist that does not induce obvious neurodegeneration during neuronal activation (Zhang et al., 1998). First, we evaluated the expression profile of Mmp-9 mRNA by RT-qPCR after bicuculline activation in the cultures. We found that Mmp-9 mRNA was significantly upregulated after neuronal activation (Figure 5A). Then, we checked whether Mmp-9 mRNA was stabilized after the activation of hippocampal neurons. We blocked mRNA synthesis by actinomycin D in the unstimulated controls, and activated hippocampal neuronal cultures (4 h after bicuculline) to calculate Mmp-9 mRNA half-life. We found that neuronal activation prolonged the half-life from 1 h and 49 min in the unstimulated cultures to 5 h and 56 min in the cultures from samples collected at 4 h after activation (Figure 5B). This indicates that the bicuculline-evoked activation induces significant Mmp-9 mRNA stabilization in the developing hippocampal neurons. Moreover, it suggests that mRNA stabilization mechanisms participate in the upregulation of neuronal Mmp-9 mRNA after the activation of hippocampal neurons.

### HuR and HuB increase Mmp-9 mRNA expression in the activated rat hippocampal neurons

**Figure 6:**
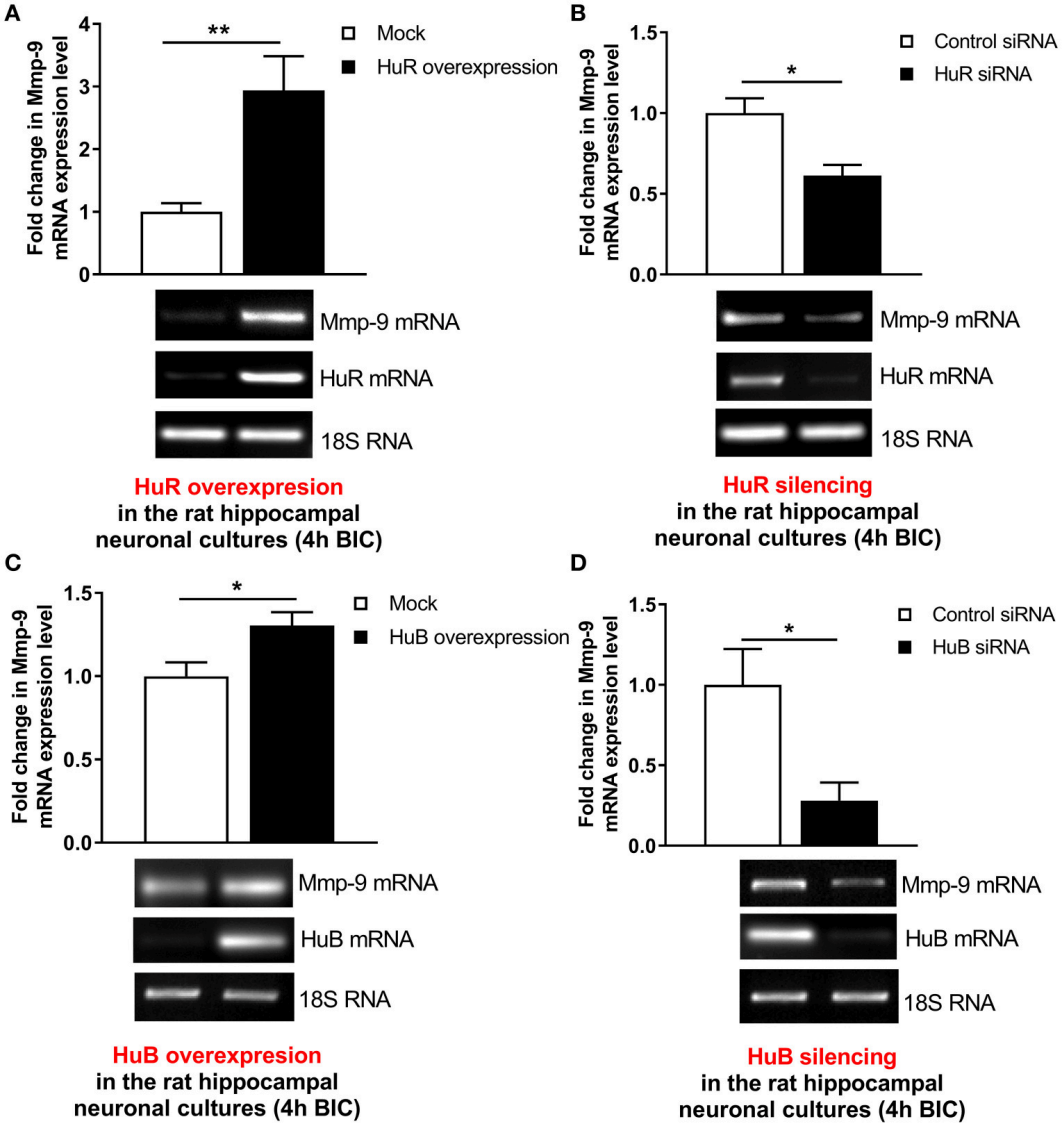
HuR and HuB upregulate Mmp-9 mRNA expression in activated rat hippocampal neurons. **(A)** HuR overexpression increases Mmp-9 mRNA in bicuculline-treated rat hippocampal neuronal cultures. The cultures were transfected by the HuR overexpression construct HuR/pGW1 or the mock control vector pGW1 using the Neon Transfection System. On Day 7 *in vitro* (DIV 7), the cultures were treated with bicuculline. mRNA content was analyzed at 4 h after neuronal activation by qRT-PCR. Mmp-9 expression was normalized against 18S rRNA expression. Data are presented as fold change in mRNA expression. Two-tailed Student's *t*-test. Values are mean ± SEM (**p < 0.01; *n* = 5). **(B)** HuR silencing downregulates Mmp-9 mRNA in bicuculline-treated rat hippocampal neuronal cultures. The cultures were transfected by HuR siRNA or control siRNA using the Neon Transfection System. On Day 7 *in vitro* (DIV 7), the cultures were treated with bicuculline. mRNA content was analyzed 4 h after neuronal activation by qRT-PCR. Mmp-9 expression was normalized against 18S rRNA expression. Data are presented as fold change in mRNA expression. Mann–Whitney *U*-test. Values are mean ± SEM (*p < 0.05; *n* = 5). **(C)** HuB overexpression increases Mmp-9 mRNA in bicuculline-treated rat hippocampal neuronal cultures. The cultures were transfected by the HuB overexpression construct HuB/pGW1 or mock control vector pGW1 using the Neon Transfection System. On Day 7 *in vitro* (DIV 7), the cultures were treated with bicuculline. mRNA content was analyzed 4 h after neuronal activation by qRT-PCR. Mmp−9 expression was normalized against 18S rRNA expression. Data are presented as fold change in mRNA expression. Two-tailed Student's *t*-test. Values are mean ± SEM (*p < 0.05; *n* = 5). **(D)** HuB silencing downregulates Mmp-9 mRNA in bicuculline-treated rat hippocampal neuronal cultures. The cultures were transfected by the HuB silencing construct HuB/pSuper or mock control vector pSuper using the Neon Transfection System. On Day 7 *in vitro* (DIV 7), the cultures were treated with bicuculline. mRNA content was analyzed 4 h after neuronal activation by qRT-PCR. Mmp-9 expression was normalized against 18S rRNA expression. Data are presented as fold change in mRNA expression. Two-tailed Student's *t*-test. Values are mean ± SEM (*p < 0.05; *n* = 5).

To confirm that Hu-dependent mRNA stabilization mechanisms can participate in the activation-evoked upregulation of neuronal Mmp-9 mRNA, we evaluated whether HuR or HuB can influence Mmp-9 mRNA expression in bicuculline-activated hippocampal neuronal cultures. We transfected the cultures with the expression construct coding for full-length rat HuR or the mock control construct, and then evaluated Mmp-9 mRNA expression by RT-qPCR (Figure 6A). Similarly, we introduced HuR siRNA or equal amounts of control siRNA into the neuronal cultures, and then measured Mmp-9 mRNA expression by RT-qPCR (Figure 6B). We found that, compared to control transfections, HuR overexpression upregulated Mmp-9 mRNA expression, whereas HuR silencing led to its clear reduction in the activated hippocampal neurons (Figures 6A,B). Then, we transfected the cultures with the expression construct coding for full-length rat HuB or the mock control construct, and thereafter evaluated Mmp-9 mRNA expression by qRT-PCR (Figure 6C). Similarly, we introduced the HuB siRNA-expressing construct or control siRNA-expressing construct into the neuronal cultures, and subsequently assessed Mmp-9 mRNA expression by RT-qPCR (Figure 6D). We observed that, compared to the control transfections, HuB overexpression upregulated Mmp-9 mRNA levels, whereas HuB silencing led to its reduction in the activated hippocampal neurons (Figures 6C,D). Furthermore, we introduced the HuC siRNA-expressing construct or control siRNA-expressing construct into the neuronal cultures, and then assessed Mmp-9 mRNA expression by RT-qPCR. We found that HuC overexpression and silencing had no effect on Mmp-9 mRNA levels in the activated hippocampal neurons (Figure S7A). Altogether, the data show that both HuR and HuB, but not HuC, specifically upregulate Mmp-9 expression in the activated hippocampal neurons. Consequently, the results suggest that the HuR- and HuB-dependent Mmp-9 mRNA stabilization mechanisms participate in the neuronal activation-triggered Mmp-9 mRNA upregulation in neurons of the hippocampus.

### HuR or HuB depletion completely blocks the occurrence of PTZ-induced Mmp-9 mRNA stabilization in the rat hippocampus

**Figure 7:**
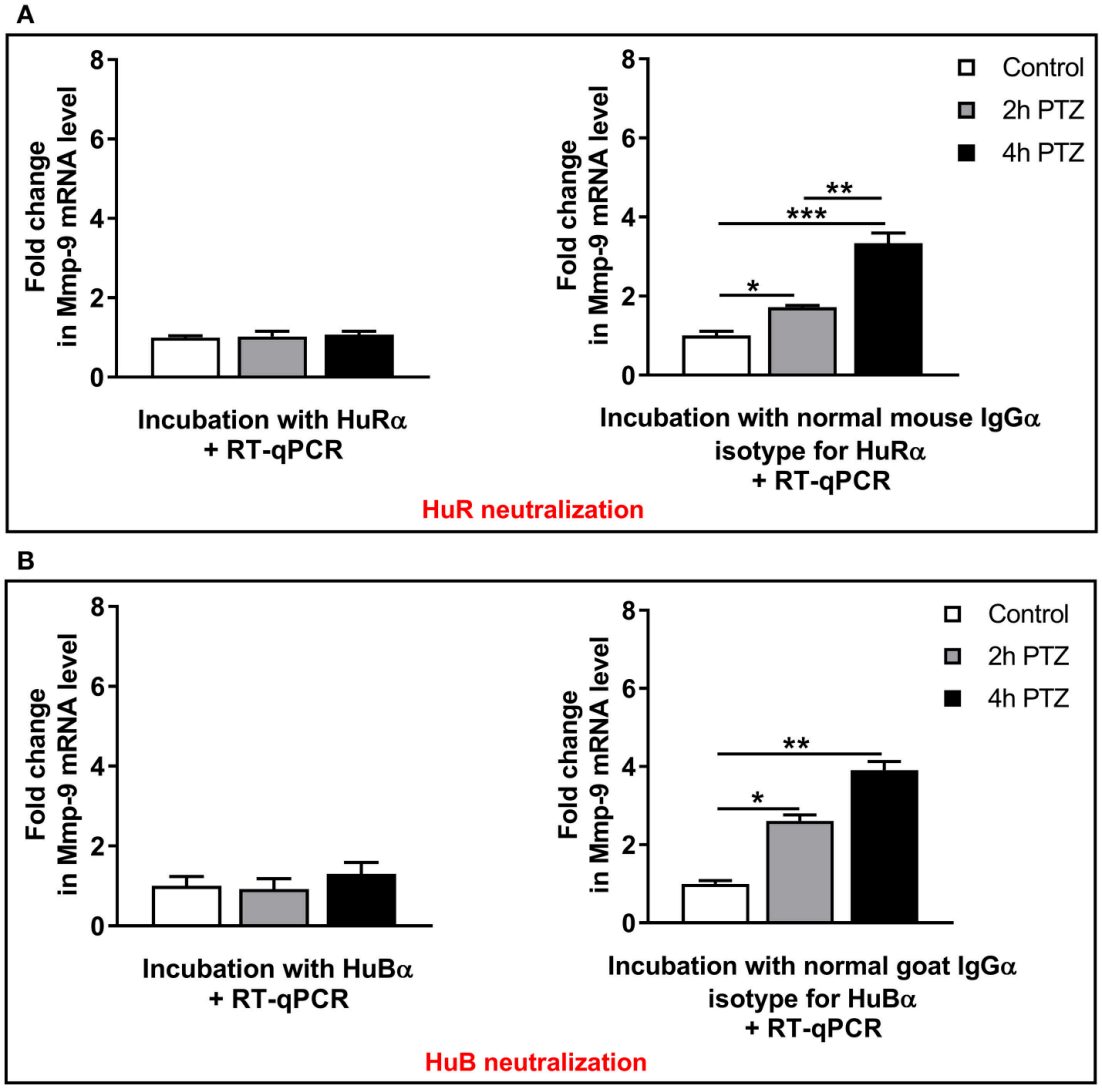
HuR or HuB depletion by immunoneutralization completely blocks the occurrence of PTZ-induced Mmp-9 mRNA stabilization in the rat hippocampus. Hippocampal cytoplasmic protein lysates (130 μg) derived from unstimulated (control) or PTZ-treated (2 and 4 h after the onset of seizures) rat hippocampi were pretreated with 0.4 μg of anti-HuR or normal mouse isotype antibody **(A)**, or anti-HuB or normal goat isotype antibody **(B)**, and then incubated with 20 μg of total RNA obtained from PTZ-treated rat hippocampi. The incubation was stopped after 60 min and the extracted RNA was evaluated for Mmp-9 mRNA expression by qRT-PCR. Data are presented as fold change in mRNA expression. One-way ANOVA was followed by Tukey's multiple comparisons test. Values are mean ± SEM (*p < 0.05; **p < 0.01; ***p < 0.001; *n* = 4).

To further reveal a functional influence exerted by HuR or HuB on Mmp-9 mRNA stabilization related to PTZ-evoked neuronal activation, we blocked their biological activities in the cytoplasm by using neutralizing anti-HuR or anti-HuB antibodies. Controls were obtained using normal isotype antibodies. To this aim, we incubated cytoplasmic lysates derived from the unstimulated as well as PTZ-activated (2 and 4 h after the onset of seizures) rat hippocampi with anti-HuR, anti-HuB, or appropriate normal isotype antibodies as well as with total RNA extracted from the rat hippocampus at 4 h after PTZ-induced seizures. After 60 min of incubation, we extracted RNA and evaluated Mmp-9 mRNA expression by RT-qPCR. We found that the neutralization of HuR or HuB completely blocks PTZ-induced Mmp-9 mRNA stabilization in the rat hippocampus, leading to strongly elevated decay of Mmp-9 mRNA (Figure 7). In contrast, in the control isotype antibody samples, Mmp-9 mRNA was increasingly stabilized after neuronal activation (Figure 7). To further confirm the specifity of HuR and HuB actions on Mmp-9 mRNA stabilization, we neutralized HuC activities, in the hippocampal cytoplasmic extracts, using anti-HuC antibody. As a result, we observed no influence of HuC neutralization on Mmp-9 mRNA stabilization after PTZ-induced neuronal activation in the hippocampus (Figure S7B). Altogether, these results clearly indicate that both HuR and HuB, but not HuC, are prerequisites for the induction of PTZ-induced stabilization of Mmp-9 mRNA in the rat hippocampus. Moreover, the data suggest that Hu-dependent Mmp-9 stabilization mechanisms are important molecular components leading to the upregulation of Mmp-9 mRNA expression after PTZ-induced neuronal activation.

## Discussion

Abnormal Mmp-9 enzymatic activity participates in the induction or progression of many disorders, including CNS diseases (Dzwonek et al., 2004). Moreover, Mmp-9 is involved in different general and cell-type–specific processes–both in neuronal and non-neuronal cells (Vafadari et al., 2016). As a consequence, mechanisms maintaining Mmp-9 mRNA expression at a physiologically appropriate level provide a substantial protective effect against its harmful actions and ensure a preservation of cell homeostasis. Furthermore, the revelation of these phenomena is important to gain an understanding of the functioning of mammalian cells, including brain neurons, under physiological and pathological conditions.

Here, we showed that the neuronal seizure-induced stabilization of Mmp-9 mRNA is one of the factors leading to its activity-driven upregulation in hippocampal neurons. Moreover, we revealed that the molecular mechanism of this stabilization is dependent on the neuronal activation-triggered, transiently increased binding of the mRNA stability-inducing protein HuR to the ARE1 and ARE4 motifs of the Mmp-9 mRNA 3′UTR as well as a stably increased association of another mRNA-stabilizing protein HuB to the ARE1 motif of the 3′UTR. Intriguingly, we demonstrated that both HuR and HuB, but not HuC, are crucial for Mmp-9 mRNA stabilization after neuronal activation.

Our results are obtained using rat model of seizure-induced neuronal activation as well as rat primary neuronal cell cultures stimulated by bicuculline. Some of our experimental data are generated using *in vitro* methods performed on *in vivo* obtained hippocampal tissues. Because the methods provide not clear *in vivo* data, we have confirmed themfurther in additional experiments. Accordingly, we have demonstrated seizure-induced stabilization of Mmp-9 mRNA by RNA degradation assay which evaluated changes in MMP-9 mRNA degradation potential of hippocampal protein lysates obtained from the hippocampi *in vivo*. Then, we have validated the finding revealing neuronal activation-triggered prolongation of Mmp-9 mRNA half-life in primary hippocampal neurons. Interactions of Hu proteins with ARE elements of the Mmp-9 mRNA 3′UTR have been revealed using REMSA and REMSA supershift methods which have demonstrated *in vitro* interactions of Hu proteins included in *in vivo* obtained hippocampal tissues with labeled RNA probes corresponding to ARE elements of the Mmp-9 mRNA 3′UTR. Next, REMSA and REMSA supershift results have been verified by experiments evaluating an influence of increased or decreased expression of Hu proteins on Mmp-9 mRNA levels in primary hippocampal neurons in culture as well as by the studies analyzing influence on Mmp-9 mRNA expression of antibody-evoked neutralization of Hu protein activities in protein extracts isolated from *in vivo* obtained rat hippocampi.

The Hu family of RNA-binding proteins is composed of ubiquitously expressed HuR, neuronally expressed HuC and HuD, as well as HuB, which is also expressed in gonads (Colombrita et al., 2013). It has been shown that HuB is differently distributed and expressed in various brain regions as compared to other neuronally expressed Hu proteins (HuC and HuD), which suggests that neuronal Hu proteins could differentially influence and regulate their target mRNAs and that HuB may have different brain functions as compared to HuC and HuD (Ohtsuka et al., 2015). In the unstimulated hippocampus, Hu proteins (including HuB and HuR) are strongly expressed and localized to the neuronal cells (Peng et al., 1998; Colombrita et al., 2013; Ohtsuka et al., 2015). It has been shown that HuC, HuD, or HuR are upregulated following repeated neuronal stimulation, such as cocaine administration, seizures, or learning (Quattrone et al., 2001; Pascale et al., 2004; Tiruchinapalli et al., 2008; Ohtsuka et al., 2015; Skliris et al., 2015). Similarly, we have demonstrated that the HuR protein and mRNA are upregulated in response to PTZ-induced neuronal activation in the hippocampus. In contrast, after kainic acid-induced seizures, HuB mRNA was stably expressed in the hippocampus once the HuB protein was downregulated, which suggested the involvement of HuB in the activity-dependent posttranscriptional regulation of gene expression in the brain (Ohtsuka et al., 2015). Our results confirm this conception. We found that HuB binding to the Mmp-9 mRNA 3′UTR as well as HuB-dependent regulation of Mmp-9 expression are neuronal activity-related phenomena, although we did not observed activity-related changes in HuB protein and mRNA expression after PTZ-evoked neuronal activation.

Preliminary evidence suggests that control of Mmp-9 mRNA abundance in brain neurons is a complex process mostly involving transcriptional mechanisms (Rylski et al., 2008, 2009). Here, we report that neuronal activity-dependent upregulation of Mmp-9 gene expression is controlled by an additional regulatory dimension, namely, mRNA stability modulation which adds to the complexity of neuronal Mmp-9 mRNA abundance control. Furthermore, our data suggest that Mmp-9 mRNA expression is subjected to the control of complex posttranscriptional gene expression mechanisms in neurons of the brain.

Regulation of Mmp-9 by HuB has never been reported. Similarly, the HuR-dependent Mmp-9 mRNA stability control in brain cells is unknown. However, it has been previously shown that Mmp-9 mRNA abundance in cells outside the CNS can be regulated by a modulation of its stability in a HuR-dependent manner (Akool et al., 2003; Huwiler et al., 2003). It has been revealed that HuR (but not HuB, HuC, or HuD) can stabilize Mmp-9 mRNA by binding to ARE1, ARE2, and ARE4 sites of its 3′UTR in the rat glomerular mesangial cells (Akool et al., 2003; Huwiler et al., 2003). Here, we showed that HuR regulates Mmp-9 mRNA stability in neurons by binding to the ARE1 and ARE4 sites of the Mmp-9 mRNA 3′UTR, but not to the ARE2 and ARE3 regions. This suggests that the pattern of Hu-dependent ARE motif-binding may reflect cell-specific differences in HuR-dependent stabilization of its target mRNAs. Mechanism of the differential, cell-specific Hu-dependent binding of ARE elements is unknown. We speculate that it might be related to different factors e.g., be a consequence of differences in intracellular signaling pathways inducing differential posttranscriptional Hu protein modifications (Matoulkova et al., 2012) or might depend on a cell-type specific conformational changes of MMP-9 mRNA (Shen and Malter, 2015).

**Figure 8:**
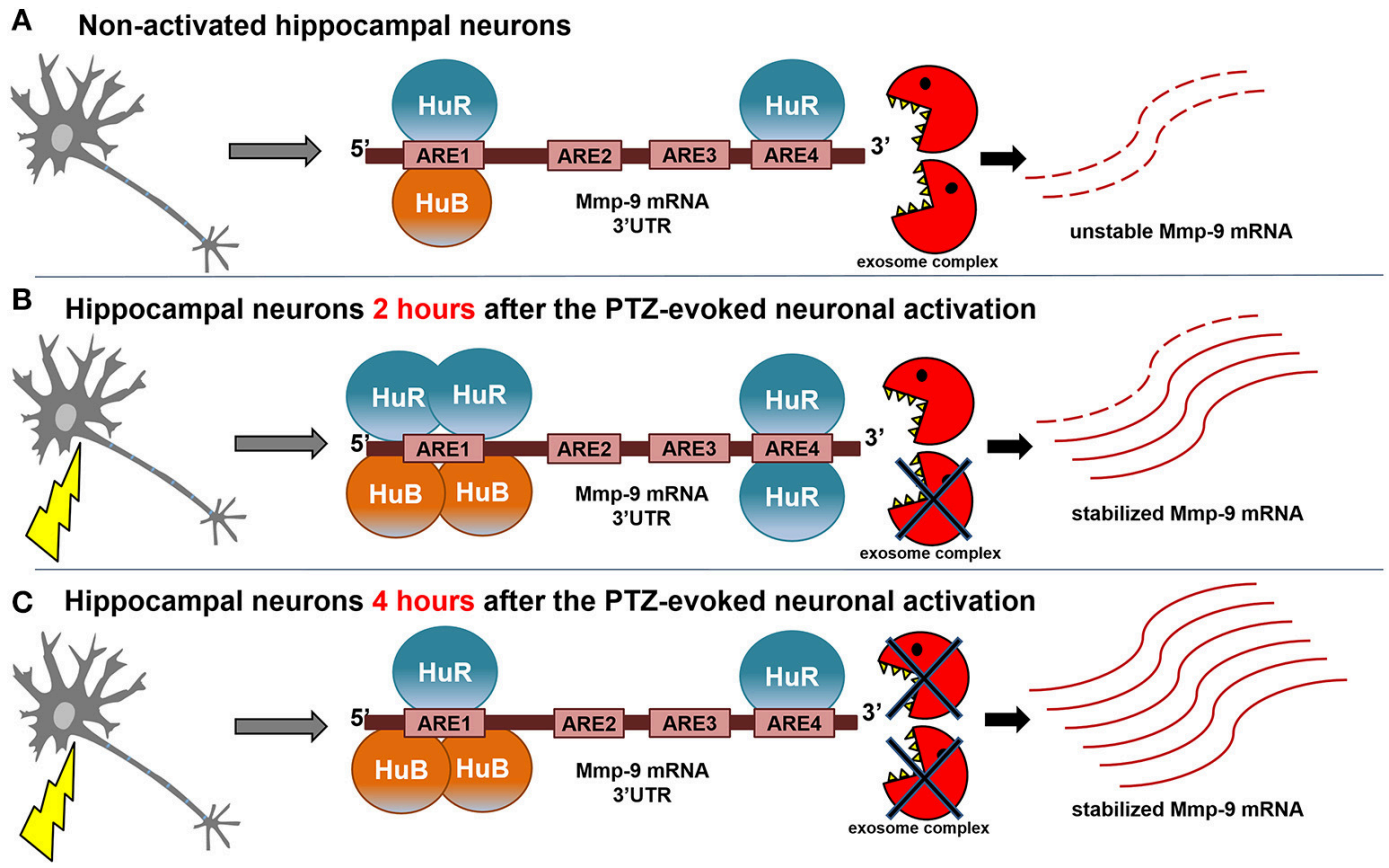
Model of neuronal activity-triggered stabilization of Mmp-9 mRNA**. (A)** In the non-depolarized hippocampal neurons, a few molecules of HuB and HuR are bound to the Mmp-9 mRNA 3′UTR (HuB is attached to ARE1 and HuR is associated with ARE1 and ARE4). As a result, Mmp-9 mRNA decay greatly enhances Mmp-9 mRNA stabilization, and Mmp-9 mRNA is expressed at low levels. **(B)** After seizure induction (2 h), there is increased binding of HuB to ARE1 as well as HuR to ARE1 and ARE4 motifs of the Mmp-9 mRNA 3′UTR. This leads to significant augmentation in Mmp-9 mRNA stability and, consequently, in its cellular abundance. **(C)** The interactions of HuR with ARE1 and ARE4 are reduced at 4 h after seizure induction, whereas HuB binding to ARE1 remains stably enhanced, which maintains elevated Mmp-9 stabilization and generates further accumulation of Mmp-9 transcripts.

Thus, mRNA stability regulation is a robust way to influence gene expression. It has been shown that even minor differences in mRNA half-life times can induce even 1,000-fold differences in a cellular abundance of individual mRNAs (Brennan and Steitz, 2001). Here, we show that Mmp-9 mRNA degradation is strongly decreased in activated hippocampal neurons (Figure 8). Moreover, we provide evidence that Mmp-9 mRNA half-life time is concurrently increased approximately 3-fold. This reveals the existence of substantial Mmp-9 mRNA stabilization, which is activated in response to neuronal stimulation. In addition, this suggests that Mmp-9 mRNA stabilization is an important component of its upregulation after neuronal activation in the hippocampus. Furthermore, this reasoning is supported by our findings that the mRNA stability inducers HuR and HuB increase their binding to the Mmp-9 mRNA 3′UTR in the neuronal activity-dependent manner (Figure 8). After PTZ-induced neuronal activation, we observed augmentation of HuR binding to ARE1 and ARE4 motifs as well as of HuB to the ARE1 site of the Mmp-9 mRNA 3′UTR. Simultaniously, we could not find HuC and HuD association to ARE elements of the 3′UTR. Moreover, we demonstrated that both HuR and HuB, but not HuC, are capable of increasing the Mmp-9 mRNA level in activated hippocampal neurons, which suggests that the Hu-dependent upregulation of Mmp-9 mRNA stability is an important regulatory mechanism promoting Mmp-9 mRNA elevation in activated hippocampal neurons. In addition, we revealed that HuR or HuB, but not HuC, depletion completely blocks the occurrence of PTZ-induced MMP-9 mRNA stabilization in the rat hippocampus. This implies that both HuR and HuB are indispensable for the existence of Mmp-9 mRNA stabilization after seizure-induced neuronal activation. Furthermore, this indicates that HuR and HuB are among the main players engaged in the regulation of *Mmp-9* gene expression in neurons. Furthermore, the data suggest that Hu-dependent Mmp-9 mRNA stability modulation can fine tune its transcriptionally induced expression or might even be responsible for the induction of transcriptional-independent quick changes in Mmp-9 mRNA content that occurs in neuronal cells in response to different stimuli.

The *Mmp-9* is a neuronal plasticity-related gene (Szklarczyk et al., 2002). Mmp-9 mRNA is expressed mostly in neurons—both in the unstimulated as well as in activated rat hippocampus (Szklarczyk et al., 2002). Neuronal activation depends on the localized translation of synaptic mRNAs. The neuronal Mmp-9 mRNA is expressed at synapses, and its synaptic level is modulated in response to neuronal depolarization (Konopacki et al., 2007; Vafadari et al., 2016). The synaptic MMP-9 mRNA participates in the regulation of the structural plasticity of dendritic spines (Vafadari et al., 2016). It was reported that the fragile X mental retardation protein (FMRP) controls dendritic localization and local translation of mRNAs, including Mmp-9 mRNA (De Rubeis and Bagni, 2010; Janusz et al., 2013). Moreover, it was shown that synaptic Mmp-9 mRNA can be directly regulated by miR-132 (Jasinska et al., 2016). Likewise, it was suggested that HuR and HuB are expressed at the synapses (Tiruchinapalli et al., 2008; Ohtsuka et al., 2015). It has been revealed that HuR regulates FMRP translation (Suhl et al., 2015), and HuB significantly affects the FMRP-targets (Berto et al., 2016). Even more intriguingly, it has been demonstrated that miRNAs can interact directly or indirectly with HuR to regulate gene expression—both positively and negatively (Shibata et al., 2011; Ahuja et al., 2016). Therefore, the available evidence suggests a possibility of the existence of Hu-dependent regulation of the synaptic Mmp-9 mRNA. Hu-dependent regulation of Mmp-9 mRNA stability is a good potential candidate mechanism to ensure the persistence of appropriate synaptic Mmp-9 mRNA content during synapse activation and in related processes such as learning and memory. HuB and HuR have a strong mechanistic molecular potential to be regulators of neuronal plasticity-related gene expression (Quattrone et al., 2001). They can postranscriptionally transform a single neuronal plasticity-related signal into a clear change in an abundance of their numerous synaptic–plasticity-related target mRNAs, leading to de novo translation and increased availability of learning and memory effector proteins, which finally elicit long-lasting synaptic changes (Quattrone et al., 2001; Tiruchinapalli et al., 2008). Accordingly, Hu proteins are strongly involved in neuronal plasticity. It has been demonstrated that neuronal Hu proteins exert learning-induced posttranscriptional control on genes encoding substrates of memory storage (Quattrone et al., 2001). Furthermore, it was shown that Hu proteins are involved in neuronal excitability control by maintaining appropriate levels of the neurotransmitter glutamate (Ince-Dunn et al., 2012). Moreover, HuB—in a cooperation with HuD—controls the expression of genes responsible for structural plasticity and shaping of neuronal projections, dendrites, and axons (Ince-Dunn et al., 2012).

Our results suggest that HuR and HuB—as the upstream Mmp-9 regulators—might be involved in gene regulation in CNS diseases related to aberrant Mmp-9 expression (e.g., epilepsy, schizophrenia, autism spectrum disorders, brain injury, stroke, neurodegeneration, brain tumors, amyotrophic lateral sclerosis). Consequently, it is well established that Hu proteins play important roles in multiple steps of neurodevelopment. It has been revealed that HuB regulates neurogenesis through engagement in the promotion of neuronal progenitor cell differentiation (Akamatsu et al., 1999; Yano et al., 2016). Moreover, HuB is involved in embryonic forebrain development (Shibata et al., 2011). Similarly, HuR is engaged in neocorticogenesis (Kraushar et al., 2014), and controls cellular energetics and oxidation metabolism in neurons, thereby conferring protection from neurodegeneration (Skliris et al., 2015). The loss of HuR sensitizes hippocampal neurons to glutamatergic toxicity (Skliris et al., 2015). HuB has been identified as a schizophrenia-related, top candidate gene in Asian populations (Yamada et al., 2011), and is involved in susceptibility to higher neuroticism (Ohi et al., 2017). Moreover, HuB regulates the expression of risk genes for autism spectrum disorder (Berto et al., 2016), and HuR is engaged in the pathogenesis of amyotrophic lateral sclerosis (Lu et al., 2014) and autism (Popovitchenko et al., 2016). Moreover, it was reported that lack of neuronal Hu proteins led to spontaneous epileptic seizure activity (Yano et al., 2016). These data demonstrate a substantial overlap in the pathological engagement of Mmp-9, HuB, and HuR and suggests a possibility of the existence of unrevealed Hu-dependent pathological potential in the pathology of other diseases related to aberrant Mmp-9 expression.

Hu proteins can regulate posttranscriptional gene expression in the brain by acting via different mechanisms, depending on the specific target mRNA and the regulatory context. The binding of HuR or ectotopically expressed HuB to ARE-containing mRNAs can result in the stabilization and increased translatability of mRNA transcripts (Jain et al., 1997; Peng et al., 1998; Kraushar et al., 2014). Interestingly, it has been shown that HuB can express both of these regulatory activities independently (Antic et al., 1999). Moreover, HuB can control neuronal activity-related gene expression by controlling alternative splicing (Berto et al., 2016). It has also been reported that Hu proteins are able to regulate mRNA polyadenylation in HeLa and, probably, in brain cells (Zhu et al., 2007). During neocorticogenesis, HuR controls a translational machinery including the phosphorylation states of initiation and elongation factors in the core translation machinery, the temporally specific positioning of functionally related mRNAs into the polysomes, and the specificity of neocortical polysomes by defining their composition (Kraushar et al., 2014); moreover, it dictates the translational specificity of target mRNAs (Popovitchenko et al., 2016). HuB-dependent mRNA stability regulation in neurons has never been reported. Genome-wide analyses examining neuronal transcriptional and splicing changes caused by HuB depletion show that HuB is an important regulator of the expression of neuronal transcripts required for the maintenance of normal neuronal and synaptic functions in the brain, and that these HuB regulatory activities are not related to the control of neuronal mRNA stability (Berto et al., 2016). Similarly, in human embryonic teratocarcinoma hNT2 cells, HuB overexpression induced neurite formation and increased direct HuB binding to the 3′UTR of the neurofilament M in mRNA, thus leading to its upregulation in a manner independent of mRNA turnover (Antic et al., 1999). However, our results described here clearly show that HuB regulates Mmp-9 mRNA abundance considerably by increasing its stability in an activation-dependent manner in hippocampal neurons. Therefore, Mmp-9 mRNA is the first HuB target in neurons regulated by mRNA stabilization. Furthermore, our results are the first to describe the existence of HuR-dependent mRNA stabilization in neurons of the brain. Such a mechanism was described so far for neuroepithelial cells undergoing mitosis (García-Domínguez et al., 2011), spinal cord motor neurons (Farooq et al., 2009; Lu et al., 2014), cerebellar neurons (Jurado et al., 2006), and non-neuronal cells (Peng et al., 1998). Intriguingly, our results show not only that HuB and HuR stabilize Mmp-9 mRNA, but also that their cytoplasmic actions are critical for the occurrence of neuronal activation-dependent Mmp-9 mRNA stabilization.

In summary, our studies define a new mechanism regulating the neuronal activity-evoked upregulation of Mmp-9 expression by acting through Hu-induced increased stabilization of Mmp-9 mRNA and, thereby, delineate a critical dependence of the stabilization on HuR and HuB actions.

## Author contributions

MR, KZ-B, and MW-G designed the study and wrote the paper. KZ-B and MW-G designed, conducted, and analyzed the experiments. MR analyzed the data. AC, MA-L, AB, and JW-D conducted and analyzed the experiments. All authors analyzed the results and approved the final version of the manuscript.

### Conflict of interest statement

The authors declare that the research was conducted in the absence of any commercial or financial relationships that could be construed as a potential conflict of interest.
