# Supplementary material for: HuR (Elavl1) and HuB (Elavl2) Stabilize Matrix Metalloproteinase-9 mRNA During Seizure-Induced Mmp-9 Expression in Neurons
Source: Front Neurosci. 2018 Apr 9;12:224. doi: 10.3389/fnins.2018.00224 (PMC5900018; doi:10.3389/fnins.2018.00224)
Supplement: Supplementary file 1 [file DataSheet1.PDF]

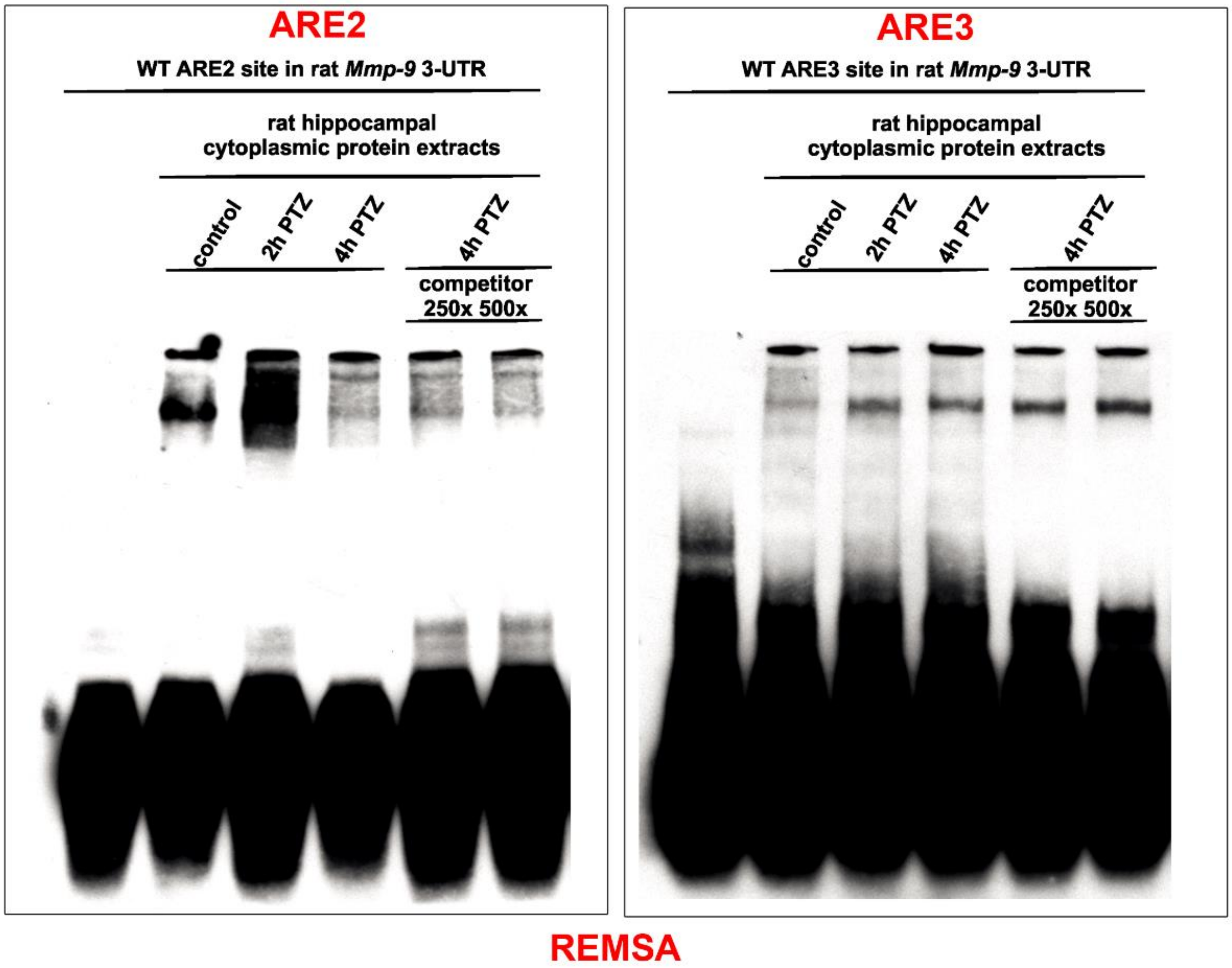

FIGURE S1. Hippocampal cytoplasmic protein complexes do not bind *in vitro* to ARE2 and ARE3 motifs of the Mmp-9 mRNA 3'UTR after the PTZ-evoked neuronal activation. The protein–RNA interactions were analyzed by REMSA. Biotinylated RNA probes derived from the ARE2 or ARE3 sites of the 3'UTR were incubated with equal amounts of cytoplasmic protein lysates isolated from the unstimulated as well as PTZ-stimulated (2 and 4 hours after PTZ-induced seizures) rat hippocampi. Additionally, as a control of binding specificity, we included samples obtained by the incubation of cytoplasmic extracts collected from the PTZ-treated (4 hours after PTZ-induced seizures) rat hippocampi with biotinylated RNA probes derived from ARE1 or ARE4 sites of the 3'UTR and mixed with 250× or 500× excess of competitors (the corresponding unlabeled ARE probes). The specifically shifted bands are marked by arrows.

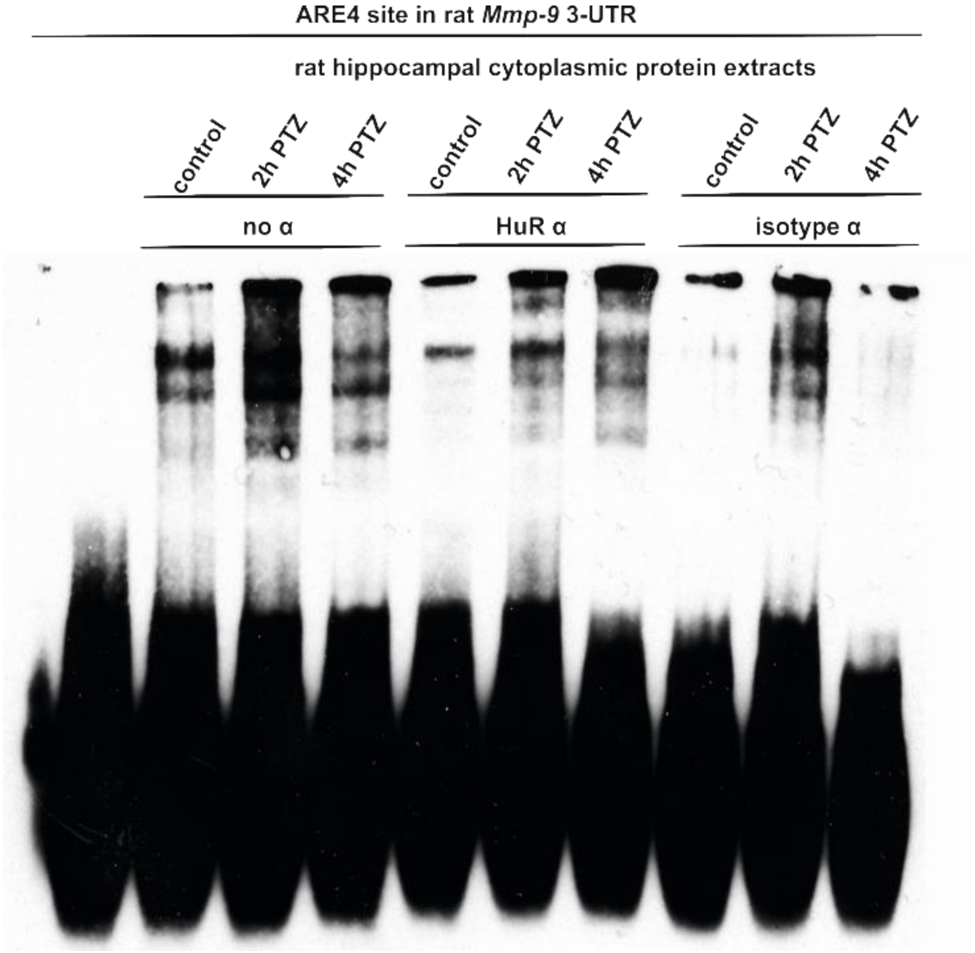

FIGURE S2. Weaker exposition of REMSA supershift blot presented on Figure 3. The interactions of HuR with Mmp-9 mRNA were studied by REMSA Supershift. Biotinylated RNA probe representing the rat Mmp-9 mRNA 3'UTR was incubated with anti-HuR antibody and with cytoplasmic protein lysates isolated from the unstimulated as well as PTZ-stimulated (2 and 4 hours after PTZ-induced seizures) rat hippocampi. In control reactions, no antibody or normal isotype antibody was added to the incubates comprising the ARE motif and cytoplasmic protein lysate.

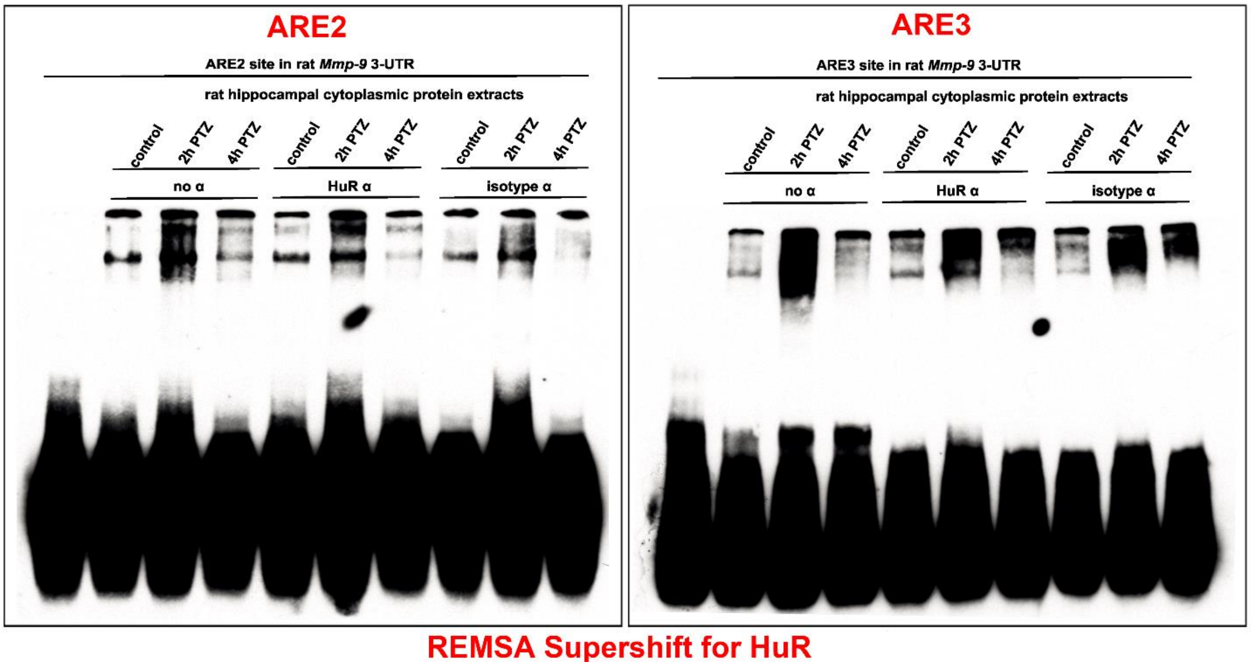

FIGURE S3. HuR does not bind to ARE2 and ARE3 motifs of the Mmp-9 mRNA 3'UTR after PTZ-evoked neuronal activation. Interactions of HuR with Mmp-9 mRNA were studied by REMSA Supershift. Biotinylated RNA probe representing ARE2 or ARE3 of the rat Mmp-9 mRNA 3'UTR was incubated with anti-HuR antibody and with cytoplasmic protein lysates isolated from the unstimulated as well as PTZ-stimulated (2 and 4 hours after PTZ-induced seizures) rat hippocampi. In control reactions, no antibody or normal isotype antibody was added to the incubates comprising ARE motif and cytoplasmic protein lysate.

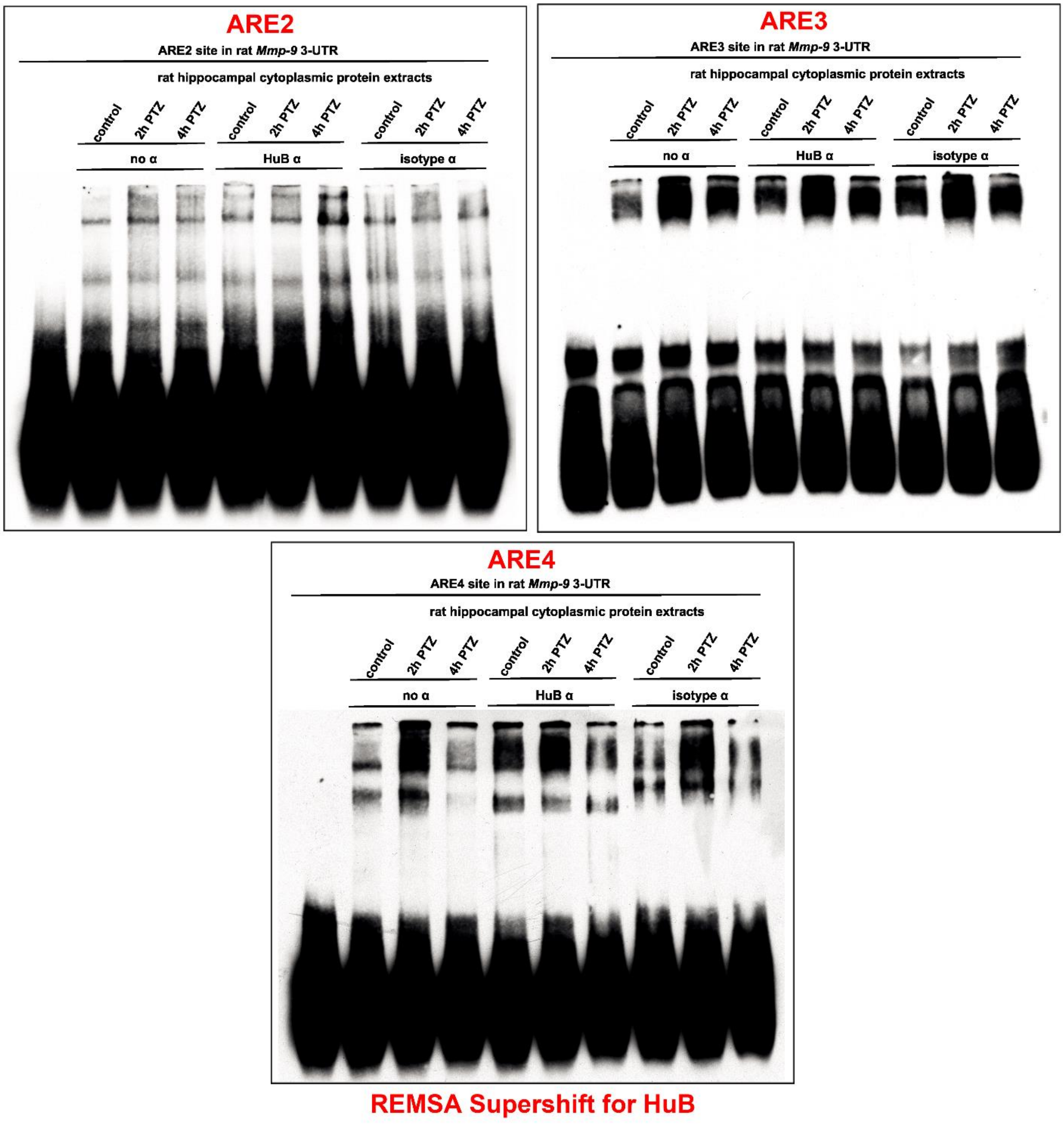

FIGURE S4. HuB does not bind *in vitro* to ARE2, ARE3, and ARE4 motifs of the Mmp-9 mRNA 3'UTR after the PTZ-evoked neuronal activation. Interactions of HuB with Mmp-9 mRNA were studied by REMSA Supershift. Biotinylated RNA probe representing ARE2, ARE3, or ARE4 of the rat Mmp-9 mRNA 3'UTR was incubated with anti-HuB antibody and with cytoplasmic protein lysates isolated from the unstimulated as well as PTZ-stimulated (2 and 4 hours after PTZ-induced seizures) rat hippocampi. In control reactions, no antibody or normal isotype antibody was added to the incubates comprising ARE motif and cytoplasmic protein lysate.

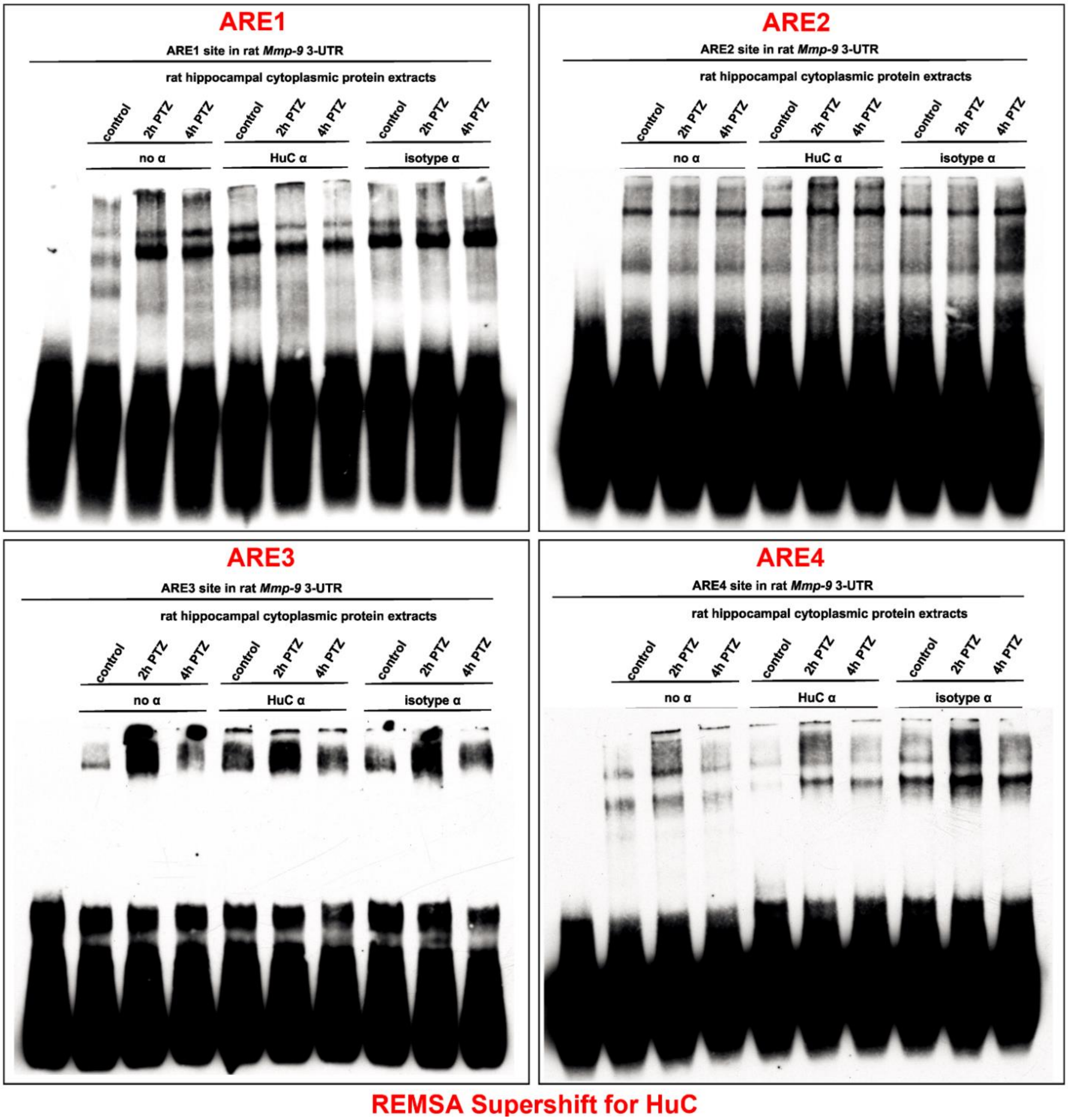

FIGURE S5. HuC does not bind to ARE1–ARE4 motifs of the rat Mmp-9 mRNA 3'UTR in the unstimulated as well as activated rat hippocampus. Interactions of HuC with Mmp-9 mRNA were studied by REMSA Supershift. Biotinylated RNA probe representing ARE1, ARE2, ARE3, or ARE4 of the rat MMP-9 mRNA 3'UTR was incubated with anti-HuC antibody and with cytoplasmic protein lysates isolated from the unstimulated as well as PTZ-stimulated (2 and 4 hours after PTZ administration) rat hippocampi. In control reactions, no antibody or normal isotype antibody was added to the incubates comprising ARE motif and cytoplasmic protein lysate.

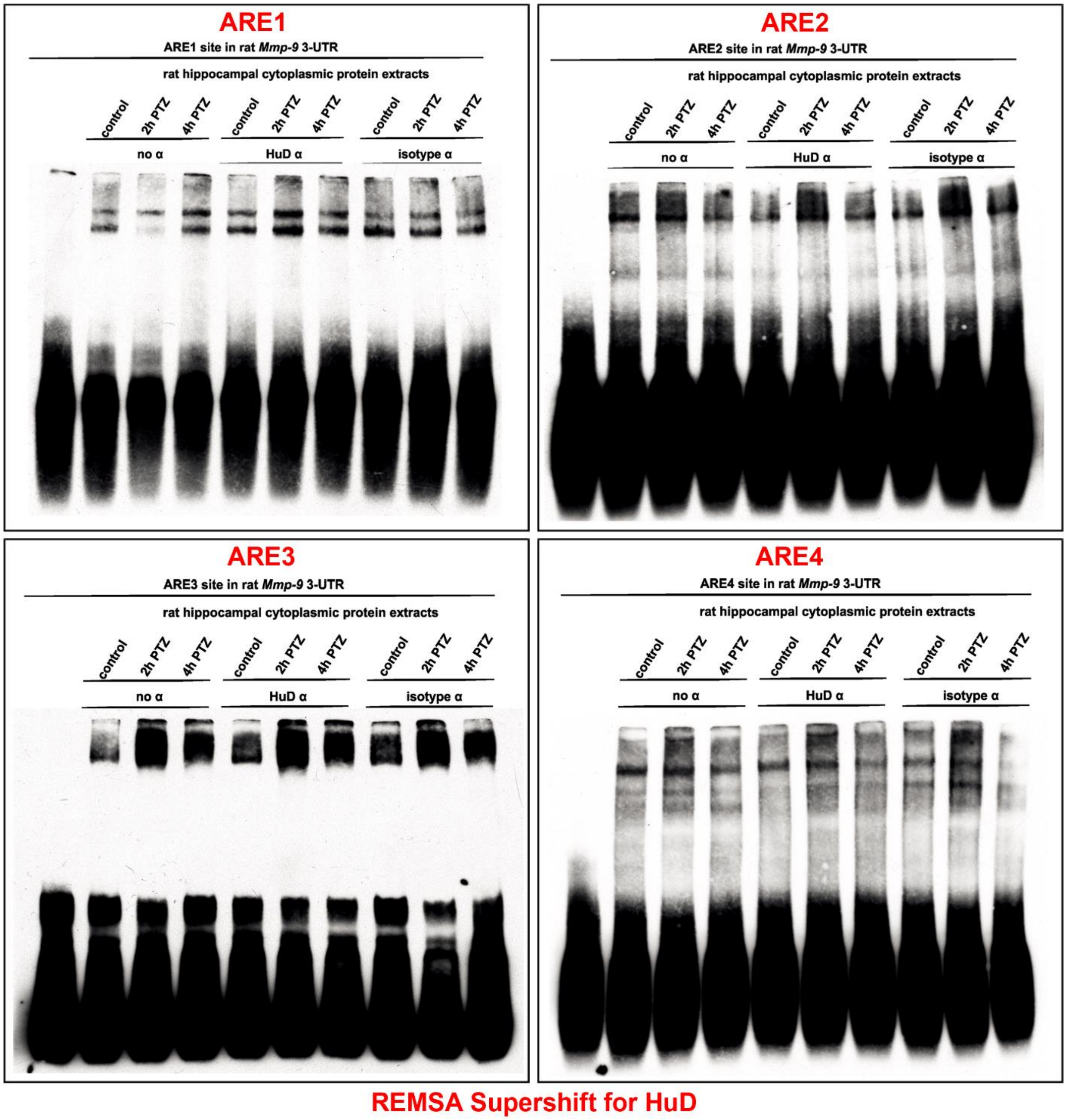

FIGURE S6. HuD does not bind to ARE1–ARE4 motifs of the rat Mmp-9 mRNA 3'UTR in the unstimulated as well as activated rat hippocampus. Interactions of HuD with Mmp-9 mRNA were studied by REMSA Supershift. Biotinylated RNA probes representing ARE1, ARE2, ARE3, or ARE4 of the rat Mmp-9 mRNA 3'UTR were incubated with anti-HuD antibody and with cytoplasmic protein lysates isolated from the unstimulated as well as PTZ-stimulated (2 and 4 hours after PTZ administration) rat hippocampi. In control reactions, no antibody or normal isotype antibody was added to the incubates comprising ARE motif and cytoplasmic protein lysate.

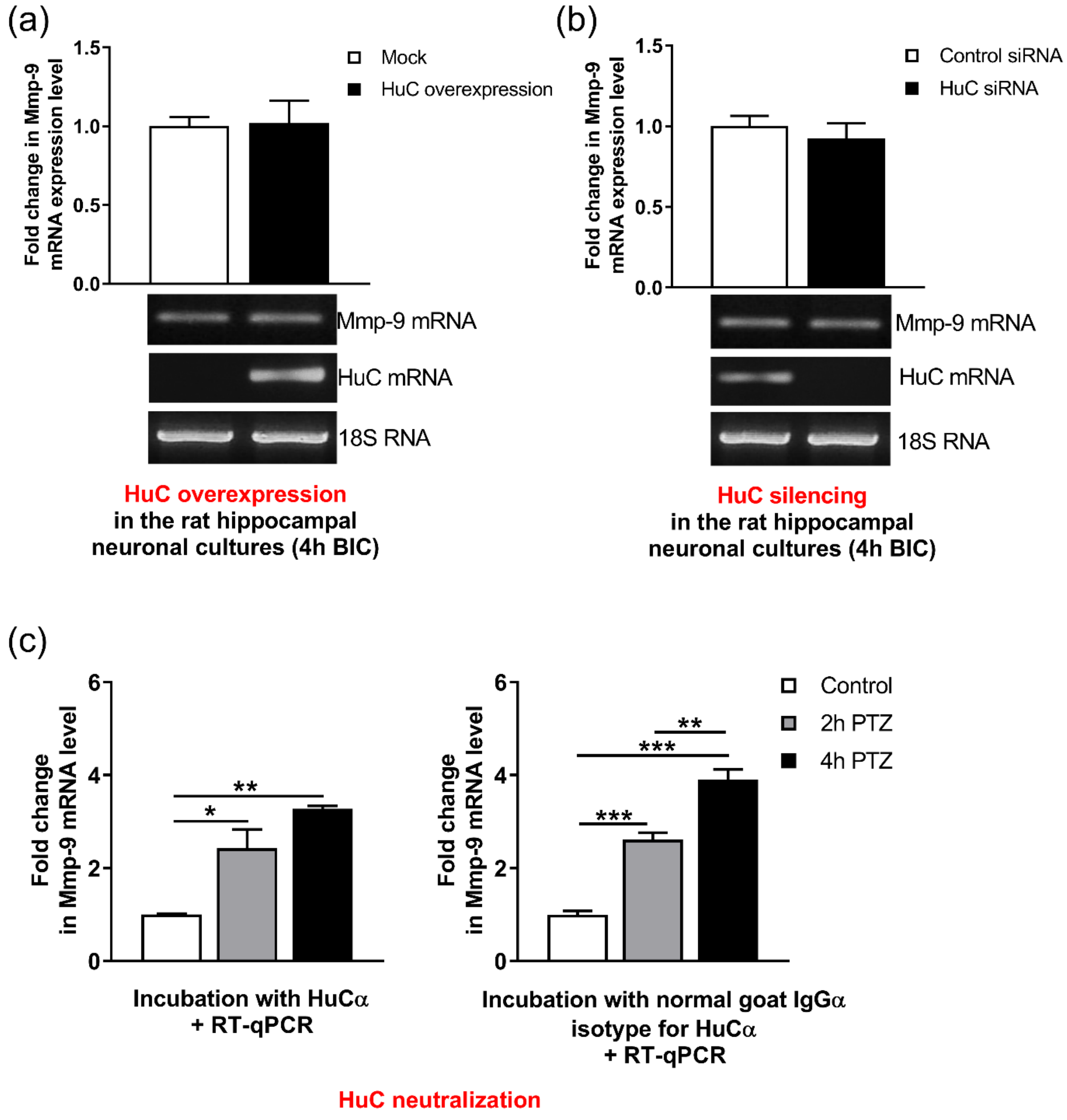

FIGURE S7. HuC does not regulate Mmp-9 mRNA expression in activated rat hippocampal neurons. (a) HuC overexpression has no effect on Mmp-9 mRNA levels in the bicuculline-treated rat hippocampal neuronal cultures. The cultures were transfected by HuC overexpression construct HuC/pGW1 or the mock control vector pGW1 using the Neon Transfection System. On Day 7 *in vitro* (DIV 7), the cultures were treated with bicuculline. mRNA content was analyzed 4 hours after neuronal activation by using qRT-PCR. Mmp-9 expression was normalized against 18S rRNA expression. Data are presented as fold change in mRNA expression. Values are mean ± SEM (n=4). **(b) HuC silencing has no influence on Mmp-9 mRNA levels in the bicuculline-treated rat hippocampal neuronal cultures.** The cultures were transfected by HuC siRNA or control siRNA using the Neon Transfection System. On Day 7 *in vitro* (DIV 7), the

cultures were treated with bicuculline. mRNA content was analyzed 4 hours after neuronal activation by qRT-PCR. Mmp-9 expression was normalized against 18S rRNA expression. Data are presented as fold change in mRNA expression. Values are mean ± SEM (*n*=4). **(c) HuC depletion by the immunoneutralization does not block the occurrence of PTZ-induced Mmp-9 mRNA stabilization in the rat hippocampus.** Hippocampal cytoplasmic protein lysates (130 µg) derived from the unstimulated (control) or PTZ-treated (2 and 4 hours after the onset of seizures) rat hippocampi were pretreated with 0.4 µg anti-HuC or normal goat isotype antibody, and then incubated with 20 µg of total RNA obtained from PTZ-treated rat hippocampi. The incubation was stopped after 60 minutes and extracted RNA was evaluated for Mmp-9 mRNA expression by qRT-PCR. Data are presented as fold change in mRNA expression. One-way ANOVA was followed by Tukey's multiple comparisons test. Values are mean ±SEM (*n*=4).
